# Acute Exposure to Cadmium Triggers NCOA4-Mediated Ferritinophagy and Ferroptosis in Never-Smokers Oral Cancer Cells

**DOI:** 10.7150/ijbs.111228

**Published:** 2025-06-20

**Authors:** Lavinia Petriaggi, Emanuele Giorgio, Stefania Bulotta, Alessandro Antonelli, Sonia Bonacci, Marialaura Frisina, Antonio Procopio, Licia Elvira Prestagiacomo, Annarita Giuliano, Marco Gaspari, Gianluca Santamaria, Giorgia Federico, Cristiana Galeano, Giuseppe Natali, Amerigo Giudice, Francesco Costanzo, Anna Martina Battaglia, Flavia Biamonte

**Affiliations:** 1 Laboratory of Biochemistry and Cell Biology, Department of Experimental and Clinical Medicine, “Magna Graecia” University of Catanzaro, Italy.; 2 Department of Health Sciences, “Magna Graecia” University of Catanzaro, Italy.; 3 School of Dentistry, Department of Health Sciences, “Magna Graecia” University of Catanzaro, Italy.; 4 Green Chemistry Laboratory, Department of Health Sciences, “Magna Graecia” University of Catanzaro, Italy.; 5 Research Centre for Advanced Biochemistry and Molecular Biology, Department of Experimental and Clinical Medicine, “Magna Graecia” University of Catanzaro, Italy.; 6 Interdepartmental Center of Services, Molecular Genomics and Pathology, “Magna Graecia” Graecia University of Catanzaro, Italy.; 7 Department of Molecular Medicine and Medical Biotechnology, University of Naples “Federico II”, Naples, Italy.

**Keywords:** Cadmium, Ferroptosis, Ferritinophagy, NCOA4, Oral Cancer, Iron Metabolism, Smokers

## Abstract

Cadmium (Cd), a carcinogenic component of tobacco, is a recognized risk factor for oral squamous cell carcinoma (OSCC). However, the molecular mechanisms underlying Cd-induced cytotoxicity in OSCC remain largely undefined. Here, we demonstrate that acute Cd exposure triggers ferroptosis in CAL27 OSCC cells derived from never-smokers, but not in SCC154 cells derived from smokers. Mechanistically, Cd outcompetes Fe, causing early iron depletion and activating the nuclear receptor coactivator 4 (NCOA4)-mediated ferritinophagy. This process enhances the labile iron pool, promotes mitochondrial reactive oxygen species (ROS) generation, lipid peroxidation, and ferroptotic cell death. Notably, iron supplementation rescues CAL27 cells from Cd-induced damage, while exacerbating iron deficiency through transferrin receptor CD71 silencing amplifies cytotoxicity. Conversely, OSCC cells from smokers exhibit resistance to Cd toxicity, likely due to the overexpression of metallothionein 2A (MT2A), a heavy metal detoxification protein. Collectively, this study provides the evidence that ferritinophagy may act as a critical upstream driver of Cd-induced ferroptosis in OSCC cells derived from never-smokers, paving the way for potential ferroptosis-targeted therapeutic strategies in Cd-associated malignancies.

## Introduction

The homeostasis of intracellular metal ions is essential for maintaining cellular integrity and function. However, disturbances in metal balance can trigger a cascade of toxic events, including oxidative damage to proteins and DNA, disruption of cell membranes, and activation of regulated cell death (RCD) pathways 1,2. In recent years, a growing body of evidence has delineated distinct forms of metals-induced RCD, namely ferroptosis, cuproptosis, and calcicoptosis, each characterized by the accumulation of specific divalent cations, namely iron (Fe²⁺), copper (Cu²⁺), and calcium (Ca²⁺), respectively 3,4. In addition, exogenous metals such as zinc (Zn²⁺), manganese (Mn²⁺), and cadmium (Cd²⁺) have been shown to induce cell death through context-dependent mechanisms, often converging on oxidative stress and mitochondrial dysfunction 5,6.

Cd^2+^ is a well-recognized environmental pollutant, primarily originating from industrial processes, agricultural activities, and tobacco-consumption 5,7-9. Although Cd^2+^ is a non-Fenton-like metal and does not directly generate reactive oxygen species (ROS), it can induce oxidative stress through several indirect mechanisms 10,11. These include (i) depletion of antioxidant molecules such as glutathione (GSH), (ii) inhibition of ROS-detoxifying enzymes, (iii) displacement of essential redox-active metals (Zn²⁺ and Fe²⁺), and (iv) impairment of mitochondrial electron transport chain, collectively resulting in mitochondrial dysfunction and ROS overproduction 10-13.

Cd²⁺-induced oxidative stress has been recognized as a key driver of several pathological conditions, including cancer 12-17. In 1993, indeed, the International Agency for Research on Cancer (IARC) classified Cd²⁺ as a Group 1 carcinogen 18. Mechanistically, Cd^2+^ exerts its carcinogenic activity through multiple pathways, including the induction of oxidative DNA damage in the form of DNA mutation, strand breaks, and chromosomal aberrations, as well as the inhibition of DNA repair systems - notably through suppression of p53 DNA binding capacity and the suppression of DNA repair-associated genes 19-21. Beyond its genotoxic effect, Cd^2+^ also exerts epigenetic effects by altering DNA and histone methylation patterns. For instance, Cd^2+^-induced hypermethylation of tumor suppressor genes promoters, such as p16, has been associated with malignant transformation of human prostate epithelial cells 22. Furthermore, chronic exposure to sub-toxic concentrations of Cd^2+^ can activate defense mechanisms against oxidative stress, including the stimulation of ROS-sensitive transcription factors, such as nuclear factor erythroid 2-related factor 2 (Nrf2), activator protein 1 (AP-1) and nuclear factor-kB (NF-kB), as well as mitogen-activated protein kinases (MAPKs)- dependent signaling pathways, which may ultimately promote cell survival and tumorigenesis 23-25.

Over time, epidemiological studies have reported a significant association between Cd²⁺ exposure and increased risk of oral squamous cell carcinoma (OSCC), the most common subtype of head and neck squamous cell carcinoma (HNSCC) 26-28. In this context, both environmental factors and genetic alterations in oncogenes and tumor suppressor genes play central roles in OSCC pathogenesis 29-33. Notably, chronic and prolonged exposure to Cd^2+^ - particularly through tobacco consumption - appears to contribute to carcinogenic transformation of the oral epithelial mucosa 9,30,34-37. However, the molecular mechanism underlying the Cd^2+^-induced toxicity in oral epithelial cells remains incompletely understood and requires further study.

In this study, we investigated the effects of Cd²⁺ acute exposure in OSCC cells derived from non-smoker and smoker patients - the latter being chronically exposed to roughly 4-5 times higher levels of Cd²⁺ compared to non-smokers. Our findings reveal that Cd²⁺ toxicity selectively affects OSCC cells derived from non-smokers whereas OSCC cells derived from smokers display resistance, likely due to the overexpression of the heavy metal detoxification protein metallothionein 2A (MT2A). Notably, we demonstrate for the first time that, in OSCC cells derived from non-smokers, ferroptosis is involved in Cd²⁺-induced cytotoxicity. Mechanistically, Cd^2+^ outcompetes Fe, thus leading to an early iron depletion, which in turn acts as a driving force for the nuclear receptor coactivator 4 (NCOA4)- mediated autophagic degradation of ferritin (ferritinohagy). Ferritinophagy, subsequently, determines an increase in labile iron pool (LIP), mitochondrial ROS production, and lipid peroxidation. Overall, this study uncovers a novel mechanism of Cd-induced cytotoxicity in OSCC cells, providing a basis for developing ferroptosis-based therapeutic strategies for Cd-associated diseases.

## Materials and Methods

### Cell lines and cell culture

Human oral squamous cell lines (OSCC) - CAL27, OT1109, SCC090, and SCC154 - were purchased from the American Type Culture Collection (ATCC, Rockville, MD, United States). CAL27 and OT1109 cells were derived from never-smoker patients, while SCC090 and SCC154 originated from tobacco users. Following ATCC instruction, CAL27 cells were grown in DMEM medium (Sigma-Aldrich, St. Louis, Missouri, United States ), while SCC154 cells were cultured in MEM (Sigma-Aldrich, St. Louis, Missouri, United States), both supplemented with 10% (v/v) fetal bovine serum (FBS) (Invitrogen, San Diego, CA), L-glutamine and 1% (v/v) penicillin and streptomycin (Sigma-Aldrich, St. Louis, Missouri, United States) at 37°C in a humidified incubator with 5% CO_2_ atmosphere. All cell lines were tested for mycoplasma contaminations and authenticated via short tandem repeat (STR) profiling.

### Reagents and treatments

Cadmium chloride (CdCl_2_), ferrostatin-1 (Fer-1) and bafilomycin (Baf) were purchased from Sigma Aldrich (Sigma-Aldrich, St. Louis, MO, USA). Ferlixit (62.5 mg/5 mL, sodium ferric gluconate complex in sucrose, SANOFI) has been obtained from the outpatient pharmacy at the Unit of Cardiology, “Magna Graecia” University of Catanzaro. Cells were seeded in a 12- and 6-well plate in complete medium. Each compound was used at the following final concentrations: CdCl_2_ at 0.1, 1, 5, 10, 50 and 100μM for 12h; CdCl_2_ at 26.1μM for 30', 1h, 6h and 12h; Fer-1 at 100μM for 24h; Baf at 1μM for 12h; ferlixit at 25, 50 and 100μM for 12h. Treatments were performed at least three times on independent biological replicates. CAL27 were exposed to 10μM CdCl_2_ for 30 days to induce metal tolerance (CAL27T); this concentration was replenished every 2-3 passages to maintain tolerance.

### Patients and clinical samples

Fourteen OSCC patients, 7 non-smokers and 7 smokers, underwent surgery at the Oral Pathology and Oral Surgery Unit of “Magna Graecia” University, between December 2020 and December 2022 32,33. For each patient, primary tumor tissue specimens were collected within the macroscopic lesion boundaries defined visually and by palpation. All patients provided a written informed consent at the time of data collection. No information that could identify individual participants are available. The procedures reported in this study were performed in accordance with the Helsinki Declaration guidelines (2008) on human experimentation and good clinical practice (good clinical practice or GCP).

### PI staining analysis

Cells were incubated with propidium iodide (PI) at 37°C for 15 min in the dark, washed twice with PBS, and analyzed using a BD LSRFortessa™ X-20 flow cytometer (BD Biosciences, San Jose, CA, USA). A total of 2×10^4^ events were acquired for each sample. Data analysis was carried out using FlowJo™ v10 Software (BD Biosciences, San Jose, CA). Each experiment was performed in triplicate.

### Apoptosis assay

Apoptotic cells populations were identified using the Alexa Fluor®488 Annexin V/Dead Cell Apoptosis Kit (Thermo Fisher Scientific, Waltham, MA, USA 38. Briefly, 1×10^5^ single-cell suspensions from CAL27 and SCC154 cell lines were centrifuged and resuspended in 100μL 1X annexin-binding buffer. To each sample, 5μL Alexa Fluor®488 Annexin V and 1μL PI working solution (100μg/mL) were added. Samples were then incubated for 15' at room temperature in the dark. Each tube was diluted with 200 μL of Annexin Binding Buffer. Flow cytometry assays were performed using the BD LSRFortessa™ X-20 (BD Biosciences, San Jose, CA, USA). Data were acquired from three independent biological replicates and analyzed out using FlowJo™ v10 Software (BD Biosciences, San Jose, CA).

### Cell viability assay (MTT)

Cell viability was assessed using the 3-[4,5-dimethylthiazolyl]-2,5-diphenyltetrazolium bromide (MTT) assay (Sigma-Aldrich, St. Louis, MO, USA) assay. Briefly, CAL27 and SCC154 cells (5 × 10^4^ cells/well) were seeded in a 24-well plates. Following exposure to CdCl_2_, cells were incubated with freshly prepared MTT solution (2 mg/mL) for 4h at 37 °C. Then, the supernatant was removed and replaced with 200μL of isopropanol to solubilize the resulting formazan crystals. Absorbance was measured at 595nm using a microplate spectrophotometer. Cell viability was expressed as a percentage relative to untreated control cells, which were set as 100%. The assay was performed at 0, 12, and 24h post-treatment. All experimental conditions were tested in triplicate across three independent experiments.

### Wound healing assay

Cells (3 × 10⁵) were seeded in 12-well plates. A scratch was introduced using a sterile pipette tip, and wound closure was monitored at 0, 12, 24, 48, and 72 h using using the Leica THUNDER Microscope DMi8 (Leica Microsystems S.r.l., Wetzlar, Germany). The gap area was quantified using by using ImageJ software. All experiments were conducted in triplicate.

### Total protein extraction and western Blot analysis

Total protein extracts were prepared using RIPA lysis buffer composed of 1M Tris HCl, Triton X-100, 3M NaCl, 0.5M EDTA, 10% SDS supplemented with cOmplete™ Protease Inhibitor Cocktail provided in EASYpacks (Roche Diagnostics, Mannheim, Germany) to prevent proteolytic degradation 39. Briefly, cells were lysed in ice-cold RIPA buffer and lysates were centrifuged at 12.000g for 30' at 4°C to remove insoluble debris. Protein concentration was determined using the Bio-Rad Protein Assay Dye according to manufacturer's instructions (Bio-Rad Laboratories, Hercules, California, United States). Equal amounts of protein (50μg) from each sample were separated by 8%-12% SDS-PAGE, run at 200V for 1h and 30'. Proteins were then transferred onto nitrocellulose membranes (Sigma-Aldrich, St. Louis, MO, United States) at 50V for 2h. Membranes were blocked with 5% non-fat milk or 5% BSA for 1h at room temperature, followed by overnight incubation at 4°C with the appropriate primary antibodies. The antibodies against ferritin heavy subunit (FtH1) (1:200, sc-376594), NCOA4 (1:500, sc-373739) and hypoxia inducible factor-1 alpha (HIF-1ɑ) (1:500, sc-10790) were purchased from Santa Cruz Biotechnology (Santa Cruz Biotechnology, Dallas, Texas, United States); antibody against glutathione peroxidase 4 (GPX4) (1:1000, ab19534) was purchased from Abcam (Abcam, Cambridge, UK), while antibodies against mechanistic target of rapamycin complex 1 (mTORC1) (1:500, 2972s), phosphorylated mTORC1 (p-mTORC1) (1:500, 5536s), microtubule associated protein 1 light chain 3B (LC3B) (1:500, #2775) and iron regulatory protein 1 (IRP1) (1:1000, 20272) were obtained from Cell Signaling Technology (Danvers, Massachusetts, United States). Membranes were washed for 30' and then incubated for 1h at room temperature with peroxidase-conjugated secondary antibodies (Peroxidase AffiniPure Sheep Anti-Mouse IgG, 1:10,000; Peroxidase AffiniPure Donkey Anti-Rabbit IgG, 1:10,000; Peroxidase AffiniPure Donkey Anti-Goat IgG, 1:10,000; Jackson ImmunoResearch Europe Ltd). Signals were detected using chemiluminescence reagents (ECL Western blotting detection system, Santa Cruz Biotechnology, Dallas, Texas) and acquired by Uvitec Alliance Mini HD9 (Uvitec Cambridge, United Kingdom). To calculate the relative expression of specific protein a mouse monoclonal IgG glyceraldehyde 3-phosphate dehydrogenase (GAPDH) HRP (1:3000; sc-47724) serves as references for sample loading. The protein band intensity on western blots was quantified and normalized to that of GAPDH by using ImageJ software.

### Transmission Electron Microscopy (TEM) for Ultrastructural Morphological Changes

CAL27 and SCC154 cells (2×10^6^ cells/well), either untreated or exposed to CdCl_2_, were centrifuged, and the resulting pellets were fixed for 3h in 3% glutaraldehyde prepared in 0.1M phosphate buffer (pH 7.4). Sample were rinsed in PBS for 15'and post-fixed in osmium tetroxide (1%) for 2h. Dehydration was performed through a graded acetone series, followed by progressive infiltration with acetone/resin mixtures and final embedding in pure Araldite resin (Fluka). Ultrathin sections (60-90 nm in thickness) were obtained using a diamond knife, mounted on copper grids (G300 Cu), and analyzed using a Jeol JEM 1400-Plus electron microscope operating at 80kV 40.

### Live-cell imaging of intracellular LIP

FerroOrange, a fluorescent probe selectively binds ferrous iron ions, was used to detect the LIP in live cells. CAL27 and SCC154 cells were seeded and treated as required. Subsequently, cells were incubated with 1μmol/L FerroOrange for 30' at 37°C. Fluorescence intensity was acquired using the Leica THUNDER Imaging Systems DMi8 (Leica Microsystems S.r.l., Wetzlar, Germany) following 12h of CdCl_2_ exposure. Each experiment was conducted in triplicate.

### Measurement of mitochondrial membrane potential and mitochondrial ROS

Changes in mitochondrial membrane potential (ΔѰ_m_) and mitochondrial ROS (mitoROS) production were measured by staining cells with TMRM (tetramethylrhodamine ethyl ester) dye (Thermo Fisher Scientific, Waltham, USA) and MitoSOX Red Mitochondrial Superoxide Indicator (Thermo Fisher Scientific Inc.), respectively. Upon treatments, cells were incubated with 5µM MitoSOX Red for 10' at 37°C for mitoROS detection, and with 100nM TMRE dye for 30' at 37°C for ΔѰ_m_ analysis. Cells were washed with PBS, centrifuged at 1000 r.p.m. for 3' and pellets were resuspended in 500μl of PBS. The analysis was performed through a FACS BD LSRFortessaTM X-20 cytofluorometer (BD Biosciences, San Jose, CA, United States). A minimum of 2×10^5^ cells was analyzed per condition. Fluorescence was measured using FlowJo™ v10 Software (BD Biosciences, San Jose, CA). Experiments were performed at least three times on independent biological replicates.

### Lipid peroxidation analysis

Lipid peroxidation was investigated through flow cytometry using BODIPY™ 581/591C11 dye (Thermo Fisher Scientific, Waltham, United States). Briefly, cells were incubated at 37°C for 30' with 2.5µM BODIPY™ 581/591C11; unincorporated dye was removed by washing twice with PBS. Oxidation of BODIPY-C11 resulted in a shift of the fluorescence emission peak from ∼590 nm to ∼510 nm proportional to lipid ROS generation. Flow cytometry assay was performed using the BD LSRFortessa™ X-20 (BD Biosciences, San Jose, CA, United States). A minimum of 2×10^5^ cells was analyzed per condition. Data analysis was carried out using FlowJo™ v10 Software (BD Biosciences, San Jose, CA). Each experiment was performed in triplicate.

### Immunofluorescence

Cells were cultured on cover slip and treated with or without CdCl_2_. Then, cells were fixed with 4% paraformaldehyde (Sigma Aldrich) and permeabilized with Triton-X-100 41. Actin filaments were stained with Alexa Fluor® 488 phalloidin (or Alexa Fluor® 555 phalloidin) (1:400, Molecular Probes, Life Technologies), while GSH was recognized with monoclonal antibody (10 µg/ml DITTA) followed by Alexa Fluor 488 (or Alexa Fluor 555) anti-mouse antibody (Molecular Probes, Thermo Fisher Scientific). Finally, cell DNA was stained with Hoechst 33258 (1µg/ml, Molecular Probes, Thermo Fisher Scientific) and observed with a Leica Stellaris confocal microscopy system (40×, 63× or 100× objective) at 1024 × 1024 resolution pixel 42.

### Inductively coupled mass spectrometer (ICP-MS) for Fe and Cd intracellular quantification

The analysis of microelements Fe and Cd in cell pellets was carried out using an ICP-MS iCAP RQ, (Thermo Fisher Scientific Inc., Bremen, Germany), equipped with a peristaltic pump and a CETAC ASX-520 auto-sampler (Thermo Scientific, Omaha, NE, USA), operating with argon gas of spectral purity (99.9995%). A tuning solution (iCAP Q/RQ Tune aqueous multielement standard solution, Thermo Scientific Bremen, Germany) was used daily to achieve mass calibration, and to maximize instrument sensitivity, resolution and ion signals, thus optimizing torch position, ion lenses, gas output, resolution axis and background. The optimal parameters are shown in Table 1.

Ultrapure water was obtained from a Milli-Q Integral 5 system (Millipore, Merck KGaA, Darmstadt, Germany). Nitric acid (HNO_3_, ≥69.0 TraceSELECT)) was purchased from Fluka analytical (Germany). Cd determination was performed by using a multielement ICP-MS calibration standards solution (IMS-102), containing 10µg mL^-1^ of As, Be, Cd, Co, Li, Ni, Se, Sr, V (Agilent, Santa Clara, California, USA). Single element analytical standards of Fe and Ca, containing 1000 µg mL^-1^ of each element, were purchased from Ultra Scientific Italia (Zedelgem, Belgium).

Sample mineralization was performed using an Anton Paar Multiwave 5000 digestion system equipped with a XF100 rotor, as reported by Cosco *et al.* 43 with some modifications. A preliminary cleaning step of PTFE vessels was carried out by adding 4mL of HNO_3_ and 4 ml of H_2_O, maintained at 1100W for 15min 44. Cell pellets were re-suspended in 5mL of ultrapure water, transferred to the vessels and digested with 3mL of nitric acid. The microwave digestion was achieved with the following operating conditions: up to 800W in 15min, hold at this power for 10 min. The mineralized samples were then collected into a graduated polypropylene test tube, diluted up to 10mL with ultrapure water, and stored at 4°C until analysis. External calibration curves were used for the microelement's quantification.

### Transferrin Receptor (*CD71*) and *NCOA4* transient knockdown

CAL27 and SCC154 cells were transfected using Lipofectamine™ 3000 Transfection Reagent (Thermo Fisher Scientific, Waltham, MA, United States) according to the manufacturer's protocol. *CD71* and *NCOA4* siRNAs were purchased from Thermo Fisher Scientific. To ensure an optimal control, cells were further transfected with Silencer™ Select Negative Control siRNA (ctrl) (Thermo Fisher Scientific, Waltham, MA, United States). The transfection efficiency was evaluated by using qRT-PCR.

### RNA isolation and comparative qRT-PCR analysis

Total RNA extraction was obtained through the Trizol RNA isolation method (Life Technologies, Carlsbad, California, United States) as previously described 45-48. All samples were DNase treated (Thermo Fisher Scientific, Waltham, Massachusetts, United States) and purity/integrity check was performed spectroscopically before use 49. Then, 1µg of total RNA was retrotranscribed using Applied Biosystems™ High-Capacity cDNA Reverse Transcription Kit (Thermo Fisher Scientific, Waltham, Massachusetts, United States). qRT-PCR was performed using the SYBR™ Green qPCR Master Mix (Thermo Fisher Scientific, Waltham, Massachusetts, United States). Analysis was performed on Applied Biosystems™ QuantStudio™ 3 (Thermo Fisher Scientific, Waltham, Massachusetts, United States). The relative mRNA expression levels were calculated through the 2^-ΔΔCT^ method and GAPDH was used as the housekeeping gene. Each experiment was performed in triplicate. Primers used for qRT-PCR are as follows: FtH1 (FW: 5'-CATCAACCGCCAGATCAAC-3', REV: 5'-GATGGCTTTCACCTGCTCA-3'); GPX4 (FW: 5'-ATCGACGGGCACATGGTTAA-3', REV: 5'-CGACGAGCTGAGTGTAGTTT-3'); MT2A (FW: 5'-CCTCCTCCAAGTCCCAGC-3', REV: 5'-CAGCAGCTTTTCTTGCAGGA-3'); HMOX1 (FW: 5'-CTTTCAGAAGGGCCAGGTGA-3', REV: 5'-CTTCACATAGCGCTGCATGG-3'); CD71 (FW: 5′-TGCTGCTTTCCCTTTCCTTG-3′, REV: 5′-GCTCGTGCCACTTTGTTCAA-3′); NCOA4 (FW: 5'-TGGAGCTTGCTATTGGTGGA-3', REV: 5'-CTGAGCCTGCTGTTGAAGTG-3'); GADPH (FW: 5′-CAAATTCCATGGCACCGTCA-3′, REV: 5′-GGCAGAGATGATGACCCTTT-3′).

### Surface CD71 analysis

Cells were incubated with an anti-CD71 antibody (anti-human CD71-PE, Catalog No. 130-099-219, Miltenyi Biotec) for 30 minutes in the dark. Following two washes with PBS (1X), cells were acquired using a BD LSRFortessaTM X-20 flow cytometer (BD Biosciences). Data analysis was performed using FlowJo™ v10 Software (BD Biosciences, San Jose, CA). Three independent experiments were carried out.

### Liquid chromatography tandem mass spectrometry (LC-MS/MS) analysis

All chemicals used in the experiments described in this section were purchased from Sigma- Aldrich unless otherwise specified. One hundred micrograms of protein extracts were diluted with RIPA buffer (150 mM NaCl, 1% Triton, 0,5% Sodium Deoxycolate, 50 mM Tris-HCl pH 8) to achieve an equal starting protein concentration (1 μg/μL) for both conditions. Subsequently, reduction and alkylation of disulfide bonds was performed by sequential addition of, respectively, 10 μL of 100 mM dithiothreitol (DDT) and 12 μL of 200 mM iodoacetamide (IAA); each step involved 1h of incubation on a Thermomixer at 37°C under gentle agitation (650 rpm). To quench residual iodoacetamide, 2 μL of 100 mM DTT was added and the reaction was allowed to proceed for 30 min at 37°C. Protein digestion was carried out according to the protein aggregation capture (PAC) protocol 1. For each tested condition, five or six technical replicates were performed. Briefly, a total of 10 μg of proteins were digested using 5 μL of MagReSyn Hydroxyl beads (100 μg of beads, Resyn Bioscienses) previously conditioned with 70% (v/v) acetonitrile (ACN). The precipitation of proteins was induced by adding pure ACN to reach a final concentration of 70% and samples were incubated in a Thermomixer at room temperature under shacking (1100 rpm) for 10 minutes. Subsequently, samples were placed on the magnetic rack and the supernatant was discarded. Beads were washed three times with 200μL of ACN and once with 200 ofμL 70% ethanol. Digestion was performed in 50 μL of 50 mM TEAB and trypsin was added at 1:50 enzyme-substrate ratio (overnight incubation at 37° C, 1100 rpm). The supernatant containing the digested peptides was harvested and beads were incubated again with 50μL of 0,1% FA (2 min at RT, 1100 rpm) to collect any residual peptides. The two supernatants were pooled together. Peptides were separated by an Easy nLC-1000 chromatographic instrument coupled to a Q- Exactive mass spectrometer (Thermo Scientific, Bremen, Germany) with a 70 min gradient time at a flow rate of 230 nl/min on a 15 cm, 75 μm i.d., in-house-made column packed with 3 μm C18 silica particles (Dr. Maisch). The gradient was generated using mobile phase A (0.1% FA, 2% ACN) and mobile phase B (0.1% FA and 80% ACN). Mobile phase B went from 4 to 24% in 35 min, from 24 to 45% in 23 min and from 45 to 100% in 5 min; the column was cleaned for 5 min with 100% of B. The DIA method consisted in a MS1 scan of 370-900 m/z at resolution of 70 000, an AGC target of 1e6 and maximum injection time of 50 ms, followed by 20 sequential MS2 windows acquired at 15 000 resolutions, with an AGC target of 1e6 and a maximum injection time of 60 ms. In detail, the 20 windows enclosed 4 windows with an isolation window of 30 m/z, 13 windows with an isolation window of 20 m/z and 3 windows with an isolation window of 50 m/z; the overlap for each window was equal to 1 m/z. Pathway enrichment analysis was performed using GSEABase 50 annotations and clusterProfiler 51. A Benjamini-Hochberg FDR cutoff of 0.05 was used for the analysis 52.

### DNA damage evaluation

DNA damage was detected by performing the cytofluorimetric analysis of cell surface phospho-γ H2A Histone Family Member X (p-γH2AX). Briefly, cells either untreated or exposed to CdCl_2_, were fixed and permeabilized using the BD Cytofix/Cytoperm™ Fixation/Permeabilization Kit (Cat. No. 554714) and stained with Phospho-Histone H2A.X (Ser139) Monoclonal Antibody (CR55T33), PE, eBioscience™ (Thermo Fisher Scientific, Waltham, Massachusetts, United States, Catalog: 12-9865-42). After washing twice with PBS (1X), cells were acquired in a FACS BD LSRFortessaTM X-20 cytofluorometer (BD Biosciences). Data were analyzed using FlowJo™ v10 Software (BD Biosciences, San Jose, CA). Three independent experiments were conducted.

### Cell cycle analysis

CAL27 and SCC154 cells (1 × 10⁶) were collected after 1 and 12h of CdCl₂ treatment, then fixed dropwise in 100% ethanol under continuous vortexing and stored at 4° C overnight. The following day, cells were rehydrated with PBS for 10' at room temperature and stained with a propidium iodide (PI) solution containing 50µg/mL PI (Sigma-Aldrich, St. Louis, MO, USA), 100 µg/mL DNase-free RNase A (Calbiochem, La Jolla, CA), and 0.01% NP-40 (USB, Cleveland, OH) in PBS. After 60' of incubation at room temperature, samples were analyzed by flow cytometry using the BD LSRFortessa™ X-20 (BD Biosciences, San Jose, CA). Data were processed with FlowJo™ v10 Software (BD Biosciences, San Jose, CA). All experiments were performed in triplicate.

### Statistics

All data were analyzed using GraphPad Prism version 10 (GraphPad Software, San Diego, CA, USA). Comparisons between two groups were performed using the unpaired Student's *t* test, while differences among multiple groups were assessed using by one-way ANOVA. A *p*-value < 0.05 was considered statistically significant. Data-Independent Acquisition (DIA) mass spectrometric data were analyzed in library-free mode by Spectronaut software, (Biognosys, version 18.4) using the default settings 2. The raw data were searched against the human database (79,684 sequences downloaded on 30 May 2022). The report output was imported in Perseus (version 2.0.6.0, Max-Planck-Gesellschaft, München) to perform statistical analysis 1. In detail, protein intensity values were transformed in the logarithmic scale (log_2_); only proteins quantified in at least four replicates of at least one sample group were kept, while missing values were imputed using default settings (width of 0.3 SD; down shift of 1.8 SD). Differentially abundant proteins between two conditions were detected by Student's t-test corrected for multiple hypothesis testing with a Permutation-based FDR equal to 0.05. An S0 value of 0.2 was used.

## Results

### Ferroptosis and autophagy contribute to CdCl_2-_ induced cytotoxicity in OSCC cells derived from never-smokers

**Figure 1:**
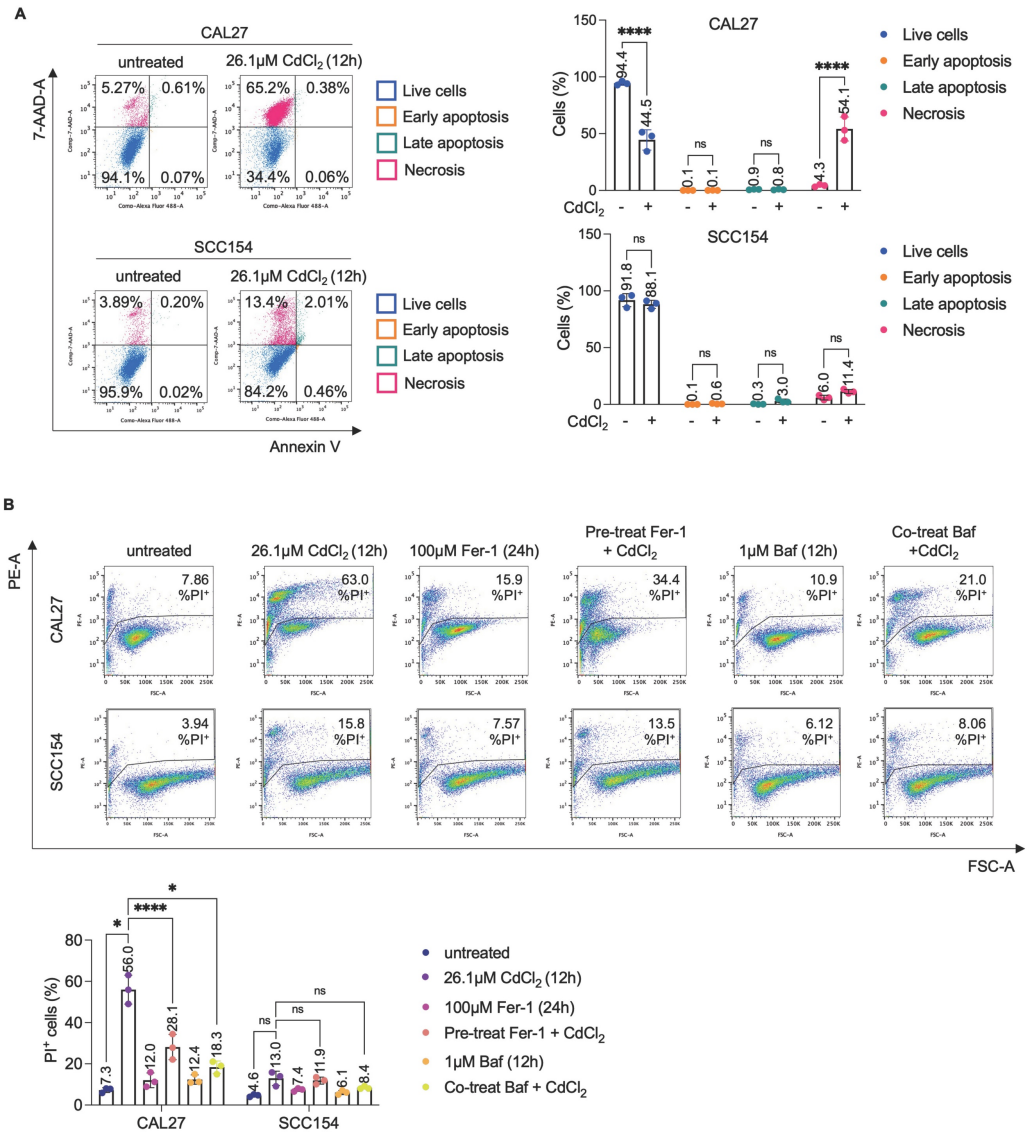
** Citotoxicity induced by CdCl_2_ is reversed by the ferroptosis and autophagy inhibitors, Fer-1 and Baf, only in CAL27 cells. (A)** Representative plots of Annexin V/7-AAD apoptosis assay (left) and relative histograms (right) of CAL27 and SCC154 cells upon treatment with 26.01μM CdCl_2_ (12h). **(B)** PI flow cytometry assay and relative histograms of CAL27 and SCC154 cells treated with CdCl_2_ (26.01μM for 12h) alone or in combination with Fer-1 (100μM for 24h) and Baf (1μM for 12h). % of dead cells (PI positive) are reported in each dot plot. All data represent the mean of three independent experiments. Histograms are reported as mean ± SD. *p*-value: *≤0.05; ****≤0.0001. ns: not significant.

Cd^2+^ exerts cytotoxic effects in cancer cells through multiple pathways, which vary according to exposure conditions - including dose, duration, and cell type specificity 9,53. Here, to investigate the impact of CdCl_2_ in OSCC, we evaluated cell viability in a panel of four OSCC cell lines: CAL27, and OT1109 (derived from never-smoker patients), SCC090 and SCC154 (derived from smokers). Dose-response analyses revealed a marked sensitivity of CAL27 and OT1109 cells to CdCl_2_ exposure (0.1 μM to 100 μM, for 12 hours), with calculated IC50 values of 26.1 μM and 74 μM, respectively. Conversely, SCC090 and SCC154 cells exhibited higher tolerance to CdCl_2_ administration across the tested concentrations Figure S1A). To elucidate the mode of cell death induced by CdCl_2_, we initially performed Annexin V/PI flow cytometry analysis. This assay excluded the involvement of apoptotic events, as no significant increase in Annexin V^+^ cells was observed following CdCl₂ treatment (Figure 1A). Then, we explored the contribution of ferroptosis and autophagy - two established forms of regulated cell death linked to oxidative stress. Pre-treatment with the ferroptosis inhibitor ferrostatin-1 (Fer-1) (100μM, 24h) or the autophagy inhibitor Bafilomycin A1 (Baf) (1μM, 12h) significantly reduced CdCl_2_-induced cytotoxicity in CAL27 cells, selected as representative of CdCl_2_- sensitive OSCC cell line, as evidenced by a decreased percentage of PI^+^ cells (Figure 1B). The protective effect of Fer-1 and Baf was not observed in SCC154 cells, a CdCl_2_- tolerant OSCC model. Furthermore, in these cells a dose- and time-dependent CdCl₂ treatment did not elicit pro-oncogenic effects, neither in terms of enhanced cell proliferation nor increased migratory capacity (Figure S1B-C). Collectively, these results demonstrate that CdCl₂ selectively induces cytotoxicity in OSCC cells from never-smoker patients, through mechanisms involving ferroptosis and autophagy activation.

### CdCl_2_ triggers NCOA4-mediated ferritinophagy in OSCC cells derived from never-smokers

**Figure 2:**
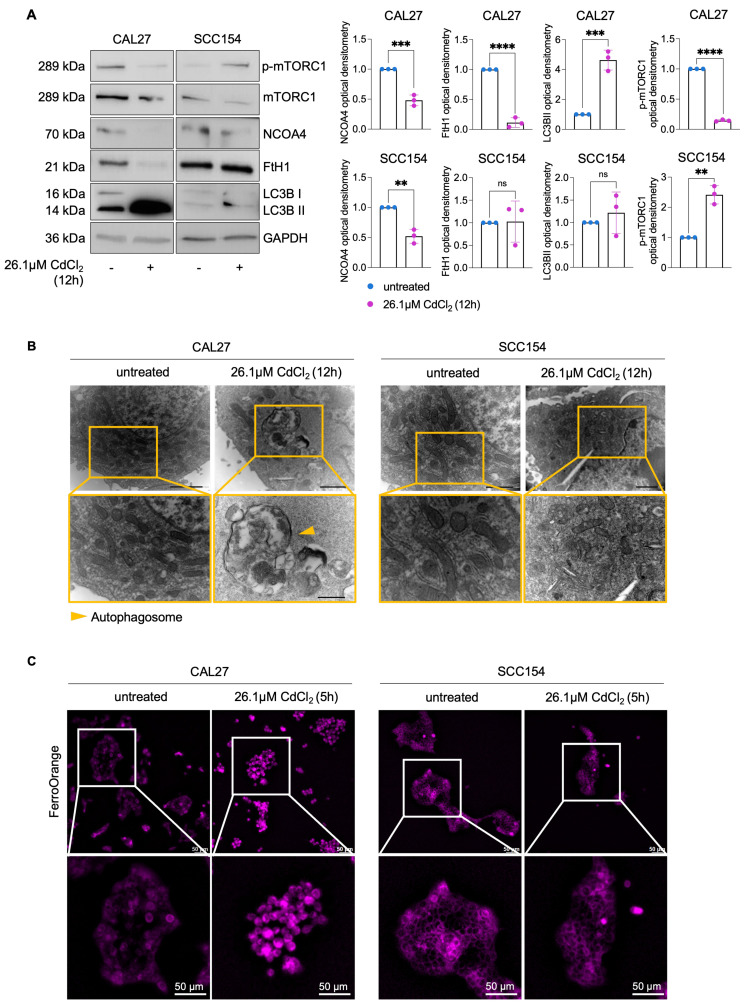
** CdCl_2_ administration induces NCOA4-mediated ferritinophagy in CAL27 cells. (A)** Western blot analysis and relative optical densitometry of NCOA4, FtH1, LC3B (I-II) and mTORC1 and p-mTORC1 in CAL27 and SCC154 cells treated with 26.01μM CdCl_2_ (12h). GAPDH was used as normalization control for protein quantification. **(B)** Representative images of morphological and ultrastructural features detected by TEM in CAL27 and SCC154 upon treatment with CdCl_2_ (26.01μM for 12h). Yellow arrows, autophagosome. **(C)** Fluorescence microscopy analysis of LIP content with FerroOrange dye in CAL27 and SCC154 cells after treatment with 26.01μM CdCl_2_ (12h). All the experiments were carried out in triplicate. Histograms are reported as mean ± SD. *p*-value: **≤0.01; ***≤0.001; ****≤0.0001. ns: not significant.

Autophagy plays a pivotal role in the execution of ferroptosis by promoting the degradation of FtH1, the main iron storage protein (54-56. This selective autophagic process, termed ferritinophagy, is orchestrated by the cargo receptor NCOA4, leading to the release of the LIP and the subsequent generation of ROS and lipid peroxidation 56,57. Whether ferritinophagy contributes to Cd^2+^-induced cytotoxicity in OSCC cells is still unknown. Here, we provide evidence that exposure to CdCl_2_ (26.1μM, 12h) markedly reduced both NCOA4 and FtH1 protein levels in CAL27. In contrast this effect was not observed in SCC154 cells, characterized by higher basal level of FtH1 compared to CAL27 cells (Figures 2A). These findings were further corroborated by the downregulation of phosphorylated mTORC1 (p-mTORC1), a master inhibitor of autophagy, coupled with the overexpression LC3B-II, a canonical marker of autophagosome formation, and the exclusive accumulation of autophagic vesicles in CAL27 cells (Figure 2A-B). Then, we monitored the real-time effect of CdCl_2_ on the intracellular LIP by using live-cell, time-lapse Leica THUNDER Imaging Systems DMi8 microscopy, with FerroOrange as a live fluorescent probe specific for ferrous iron (Fe^2+^). As shown in Movies S1-S4 and the representative frame in Figure 2C, CdCl_2_ exposure induced a substantial increase in the LIP after approximately 5h, which persisted for up to 8h in CAL27 cells.

### Mitochondrial ROS drive ferroptosis triggered by CdCl_2_ in OSCC cells derived from never-smokers

**Figure 3:**
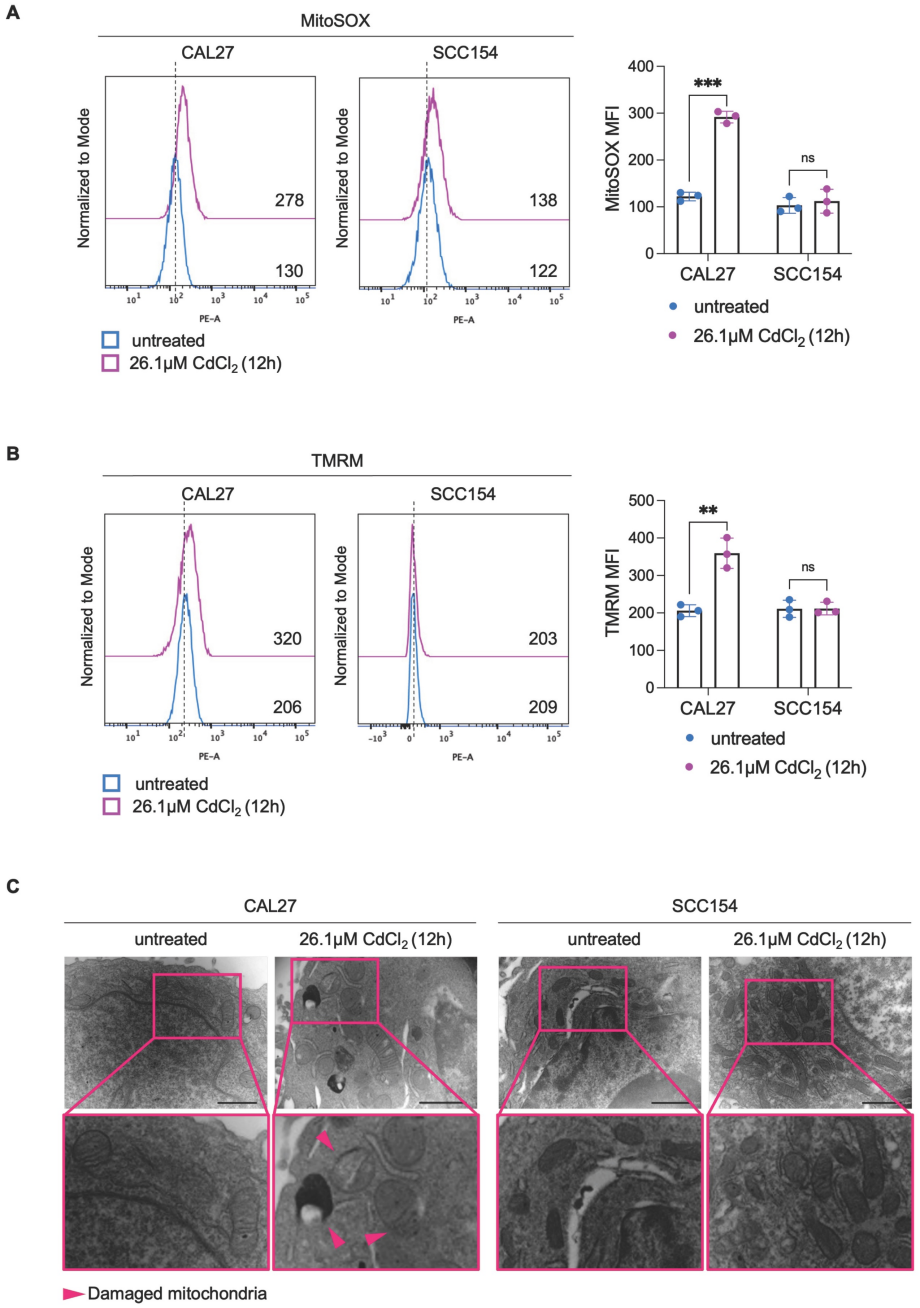
** CdCl_2_ treatment triggers mitochondrial dysfunction only in CAL27 cells.** Flow cytometry analyses and relative histograms of mitochondrial ROS levels **(A)** and mitochondrial membrane potential **(B)** assessed by MitoSOX and TMRM reagents, respectively, in CAL27 and SCC154 cells following treatment with 26.01μM CdCl_2_ (12h). **(C)** Representative images of morphological and ultrastructural features detected by TEM in CAL27 and SCC154 upon treatment with CdCl_2_ (26.01μM for 12h). Pink arrows: damaged mitochondria. Each experiment was performed in triplicate. Histograms are presented as mean ± SD. *p*-value: ***≤0.001; ****≤0.0001. ns: not significant.

**Figure 4:**
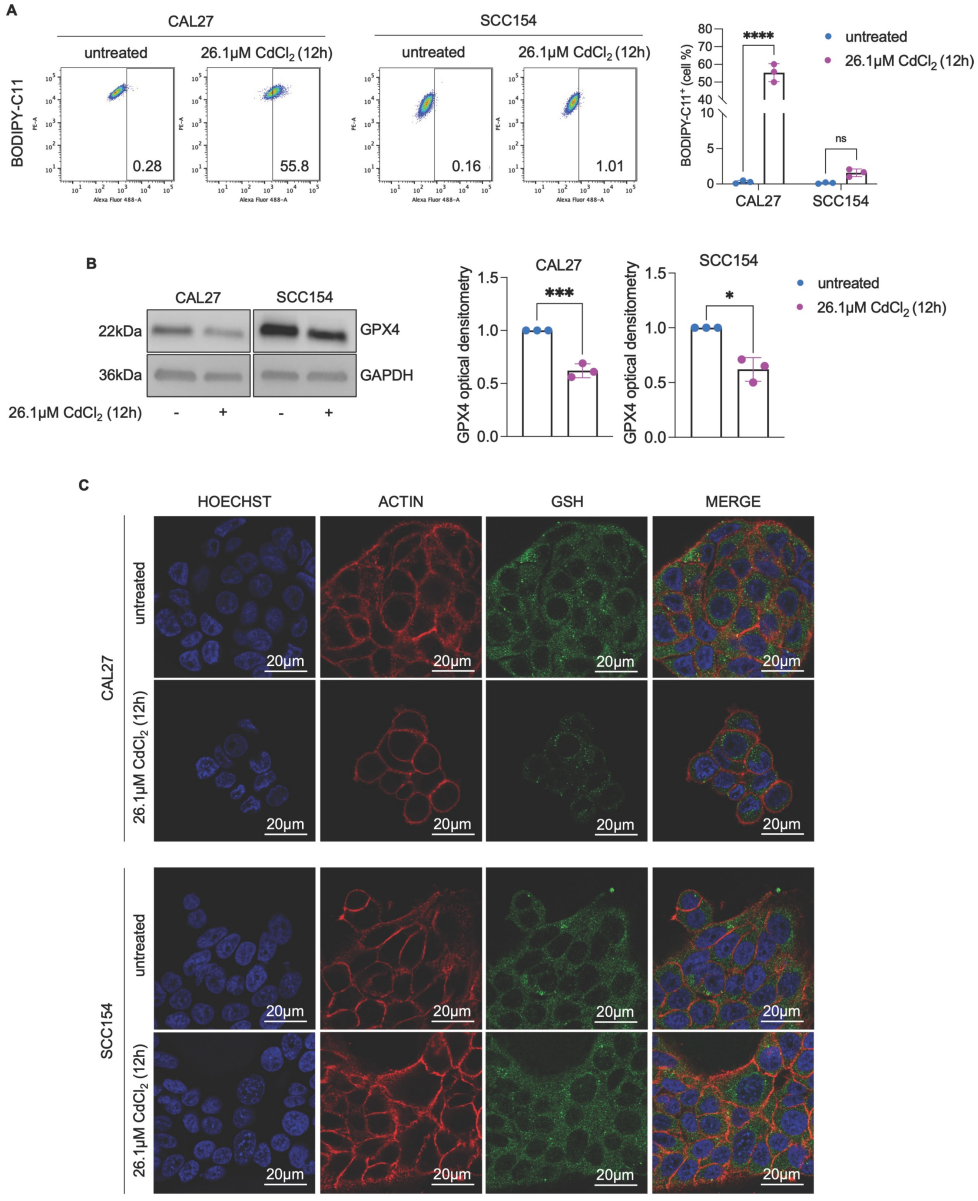
** CAL27 cells shows lipid peroxidation after CdCl_2_ administration. (A)** Flow cytometry analysis and relative histograms of lipid peroxidation quantified by using BODIPY-C11 in CAL27 and SCC154 cells upon treatment with 26.01μM CdCl_2_ (12h). **(B)** Western blot analysis and relative optical densitometry of GPX4 in CAL27 and SCC154 cells treated with 26.01μM CdCl_2_ (12h). GAPDH was used as normalization control for protein quantification. **(C)** Fluorescence microscopy analysis of GSH content in CAL27 and SCC154 cells after treatment with 26.01μM CdCl_2_ (12h). ACTIN and DAPI dyes were used to detect microfilament and nuclei, respectively. Scale bar: 20 μM. All data represent the mean of three independent experiments. Histograms are reported as mean ± SD. *p*-value: *≤0.05; ***≤0.001; ****≤0.0001.

Mitochondrial metabolism and function are deeply perturbed during ferroptosis, contributing to the amplification of oxidative stress and cell death 58-60. However, whether mitoROS are implicated in Cd^2+^-induced ferroptosis in OSCC cells remains largely unexplored. To address this question, we first assessed mitoROS generation after treating CAL27 and SCC154 cells with 26.1 μM CdCl_2_ for 12h. Flow cytometry analysis using MitoSOX^TM^ revealed that CdCl_2_ causes roughly a 2-fold increase in mitoROS levels in CAL27 (MFI, CAL27^untreated^: 130 vs. CAL27^26.1μM CdCl2^: 278, *p*-value ≤ 0.001) whereas no significant changes were observed in SCC154 cells (MFI, SCC154^untreated^: 122 vs. SCC154^26.1μM CdCl2^: 138) (Figure 3A). In agreement, we detected a marked increase in ΔѰ_m_ (TMRM MFI, CAL27^untreated^: 206 vs. CAL27^26.1μM CdCl2^: 320, *p*-value ≤ 0.001) along with the appearance of ultrastructural mitochondrial changes, indicative of mitochondrial dysfunction in CAL27 cells (Figure 3B-C). CdCl_2_ exposure strongly induced lipid peroxidation in CAL27, as demonstrated by a dramatic increase in C11-BODIPY^+^ cells compared to negative controls (CAL27^untreated^: 0.28% vs. CAL27^26.1μM CdCl2^: 55.8%, *p*-value ≤ 0.0001) (Figure 4A). This was accompanied by a downregulation of GPX4 and GSH levels (Figure 4B-C), indicating a profound impairment of the antioxidant defense system in CAL27 cells. It leaps to the eye that, as observed for FtH1, the basal levels of the antioxidant enzyme GPX4 are higher in SCC154 cells, derived from smokers, than in CAL27 derived from never-smokers. Moreover, although CdCl_2_ treatment led to GPX4 downregulation, GSH levels remained largely unaltered, suggesting the maintenance of the antioxidant homeostasis and a potential mechanism underlying CdCl_2_ tolerance in SCC154 cells. Finally, to confirm the role of ferroptosis in CdCl_2_-induced cell death, we examined the effect of Fer-1 and Baf on mitochondrial dysfunction and lipid peroxidation. As shown in Figure S2 and S3, Baf - but not Fer-1, effectively attenuated CdCl_2_-induced mitoROS production and mitochondrial membrane hyperpolarization; both Fer-1 and Baf strongly inhibited CdCl_2_- triggered lipid peroxidation 58,59.

### Early iron depletion triggers ferritinophagy-mediated ferroptosis in CdCl_2_ -treated OSCC cells derived from never-smokers

**Figure 5:**
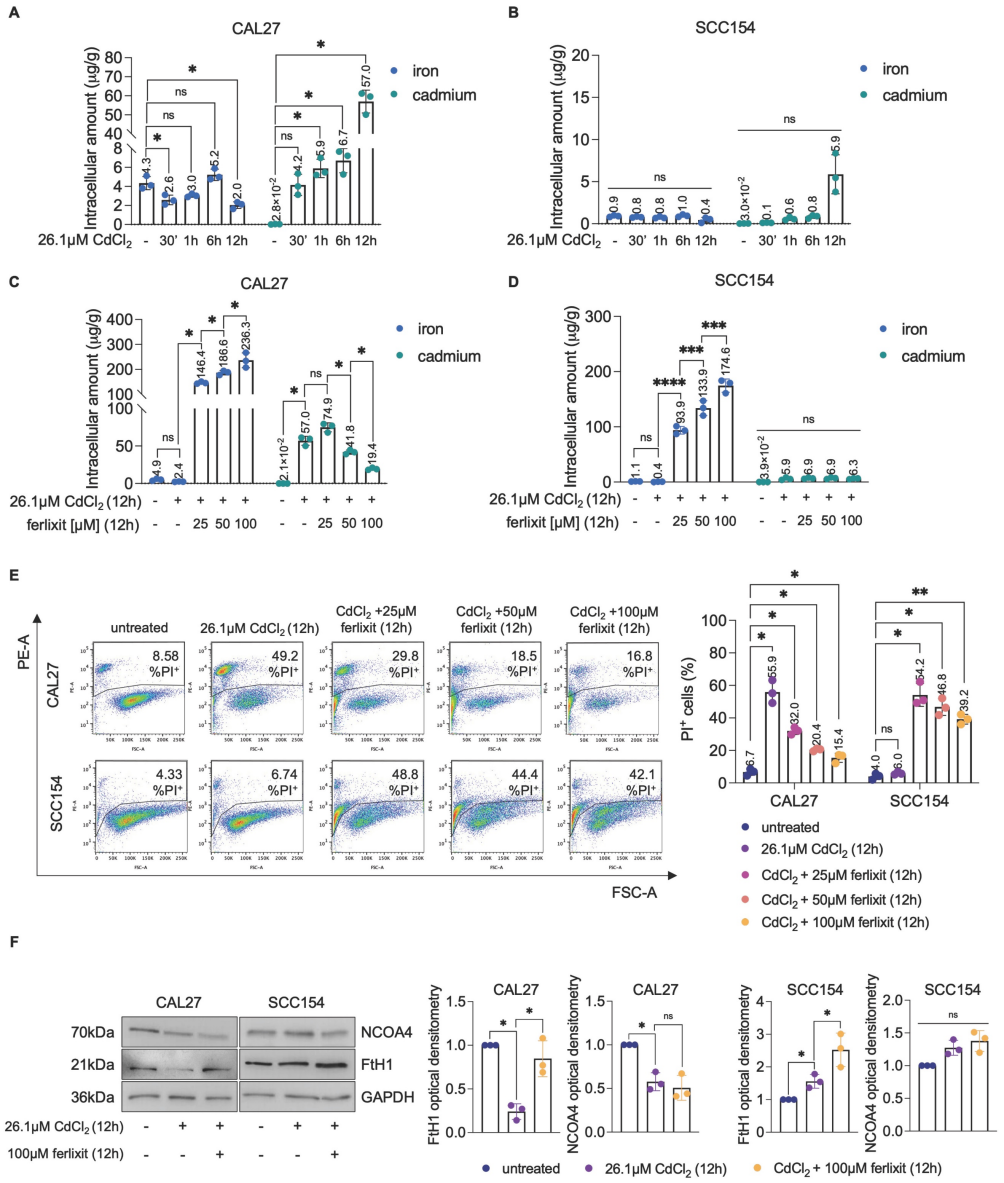
** CdCl_2_ citotoxicity is associated with the ability of cadmium to compete with iron. (A-B).** Quantification of iron and cadmium intracellular amount through ICP-MS in CAL27 and SCC154 cells treated with 26.01μM CdCl_2_ at 30', 1h, 6h, and 12h. **(C-D)**. ICP-MS analysis of iron and cadmium intracellular amount in CAL27 and SCC154 cells upon treatment with 26.01μM CdCl_2_ (12h) alone or in combination with ferlixit (25, 50 and 100μM for 12h). **(E)** PI flow cytometry assay and relative histograms of CAL27 and SCC154 cells treated with CdCl_2_ (26.01μM for 12h) alone or in combination with ferlixit (25, 50 and 100 μM for 12h). % of dead cells (PI positive) are reported in each dot plot. **(F)** Western blot analysis and relative optical densitometry of NCOA4 and FtH1 in CAL27 and SCC154 cells treated with 26.01μM CdCl_2_ (12h) alone or in combination with 100μM ferlixit (12h). GAPDH was used as normalization control for protein quantification. All data represent the mean of three independent experiments. Histograms are reported as mean ± SD. *p*-value: *≤0.05; **≤0.01; ***≤0.001; ****≤0.0001. ns: not significant.

A large part of Cd^2+^ toxicity has been attributed to its ability to compete with other essential metals, particularly iron 61,62. Cd^2+^ can outcompete iron during cellular uptake, leading to iron deficiency 62,63. Interestingly, ferritinophagy is considered a physiological response to cellular iron deficiency or starvation, leading to the release of large amounts of Fe^2+^ 64-66. Based on this evidence, we hypothesized that the NCOA4-mediated ferritinophagy observed in CAL27 cells upon 12h exposure to CdCl_2_ might be due to an early dysregulation of intracellular iron homeostasis. Hence, we quantified total intracellular Cd and Fe content in CAL27 and SCC154 cells at 30 min, 1h, 6h, and 12h exposure to 26.1μM CdCl_2_ by using ICP-MS. As shown in Figure 5A-B, although both cell lines exhibited comparable basal Cd levels (mean content, CAL27: 2.8 x 10^-2^ μg/g* vs* SCC154: 3.0 x 10^-2^ μg/g), CAL27 cells showed a pronounced and time-dependent accumulation of intracellular Cd, reaching 57.0 μg/g at 12h; conversely, intracellular Cd levels in SCC154 cells, remained substantially low, not exceeding the mean of 5.9 μg/g. Notably, as intracellular Cd levels increased, CAL27 cells underwent an early intracellular Fe depletion at 30 min (mean content, 4.3μg/g* vs* 2.6μg/g, *p*-value ≤ 0.05), followed by a transient recovery at 6h (mean content, 5.2μg/g), and a subsequent reduction at 12h (mean content, 2.0 μg/g). No significant changes in total intracellular Fe levels were observed in SCC154 cells (Figures 5A-B). These findings highlighted a striking difference in Cd accumulation capacity between the two OSCC cell lines and suggested that Cd^2+^ competes with Fe in CAL27 but not in SCC154 cells. To validate this hypothesis, we co-treated CAL27 and SCC154 cells with CdCl_2_ and increasing concentrations of ferlixit (Fe^3+^, 25μM, 50 μM, 100 μM) for 12h. Notably, in CAL27 cells, ferlixit supplementation resulted in a dose-dependent reduction of intracellular Cd (mean Cd content, CAL27^CdCl2+25μMferlixit^: 74.9μg/g, CAL27^CdCl2+50μMferlixit^: 41.8μg/g, CAL27^CdCl2+100μMferlixit^: 19.4μg/g, p-value ≤ 0.05; mean Fe content, CAL27^CdCl2+25μMferlixit^: 146.4μg/g, CAL27^CdCl2+50μMferlixit^: 186.6μg/g, CAL27^CdCl2+100μMferlixit^: 236.3μg/g, *p*-value ≤ 0.05) (Figures 5C) and a consequent reduction of cell death (Figure 5E). In contrast, in SCC154, iron supplementation did not alter intracellular Cd levels, but rather caused roughly 40-50% mortality (Figure 5D-E). Besides, ferlixit prevented the activation of ferritinophagy in CAL27 cells, as evidenced by the decrease of NCOA4 and the concomitant restoration of FtH1 (Figure 5F). Then, to assess the role of ferritinophagy in CdCl_2_-induced ferroptosis, we performed a transient knockdown of *NCOA4* Figure S4A). As reported in Figure S4B-C, although NCOA4 silencing restored FtH1 levels of CAL27 cells treated with CdCl_2_, it did not result in any significant change in cell death rates. This lack of effect can likely be attributed to a compensatory mechanism activated by CAL27 cells in response to iron depletion, involving the activation of IRP1 and the subsequent upregulation of the major iron uptake protein, CD71 (Figure S4B). Diversely, we found that transiently knocking down *CD71* and blocking Fe intake exacerbated the cytotoxic effects of CdCl_2_ at 12h in CAL27 cells (Figure S4D-E). Collectively, these findings demonstrate a differential capacity for Cd accumulation between CAL27 and SCC154 cells; it appears that Cd outcompete Fe, leading to early iron depletion that may serve as the initiating trigger for ferritinophagy-mediated ferroptosis in OSCC cells derived from never-smokers.

### Proteomic analysis reveals altered expression of proteins involved in iron homeostasis, hypoxia, and cell death in CdCl_2_ - treated OSCC cells derived from never-smokers

**Figure 6:**
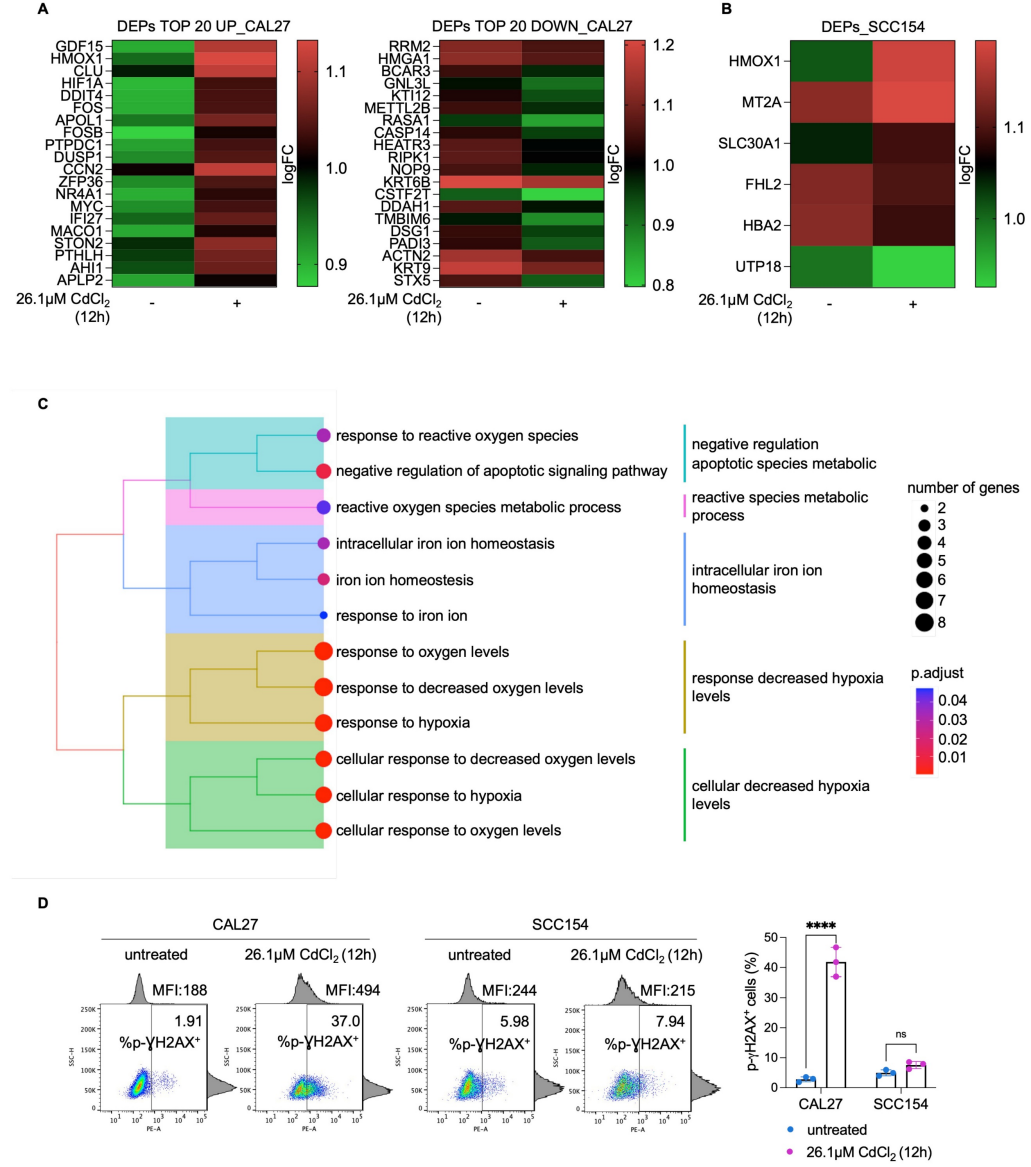
** Proteomic analysis of OSCC cells treated with CdCl_2_. (A)** Heatmap of DEPs (TOP 20 up and TOP 20 down) in CAL27 cells treated with 26.01μM CdCl_2_ (12h) vs untreated. Color intensity is proportional to the magnitude of changes. Relative expression levels are shown in red (upregulation) and green (downregulation).** (B)** Heatmap of DEPs in SCC154 cells treated with 26.01μM CdCl_2_ (12h) *vs* untreated. Color intensity is proportional to the magnitude of changes. Relative expression levels are shown in red (upregulation) and green (downregulation).** (C)** GO analysis of of DEPs in CAL27 cells treated with 26.01μM CdCl_2_ (12h) *vs* untreated. The dot size denotes the number of DEPs, while colors correspond to the adjusted *p*-value range. **(D)** p-ƔH2AX flow cytometry analysis and relative histograms CAL27 and SCC154 cells treated with CdCl_2_ (26.01μM for 12h). % of positive (^+^) cells are reported in each dot plot. Each experiment was performed in triplicate. Histograms are presented as mean ± SD. *p*-value: ****≤0.0001. ns: not significant.

**Figure 7:**
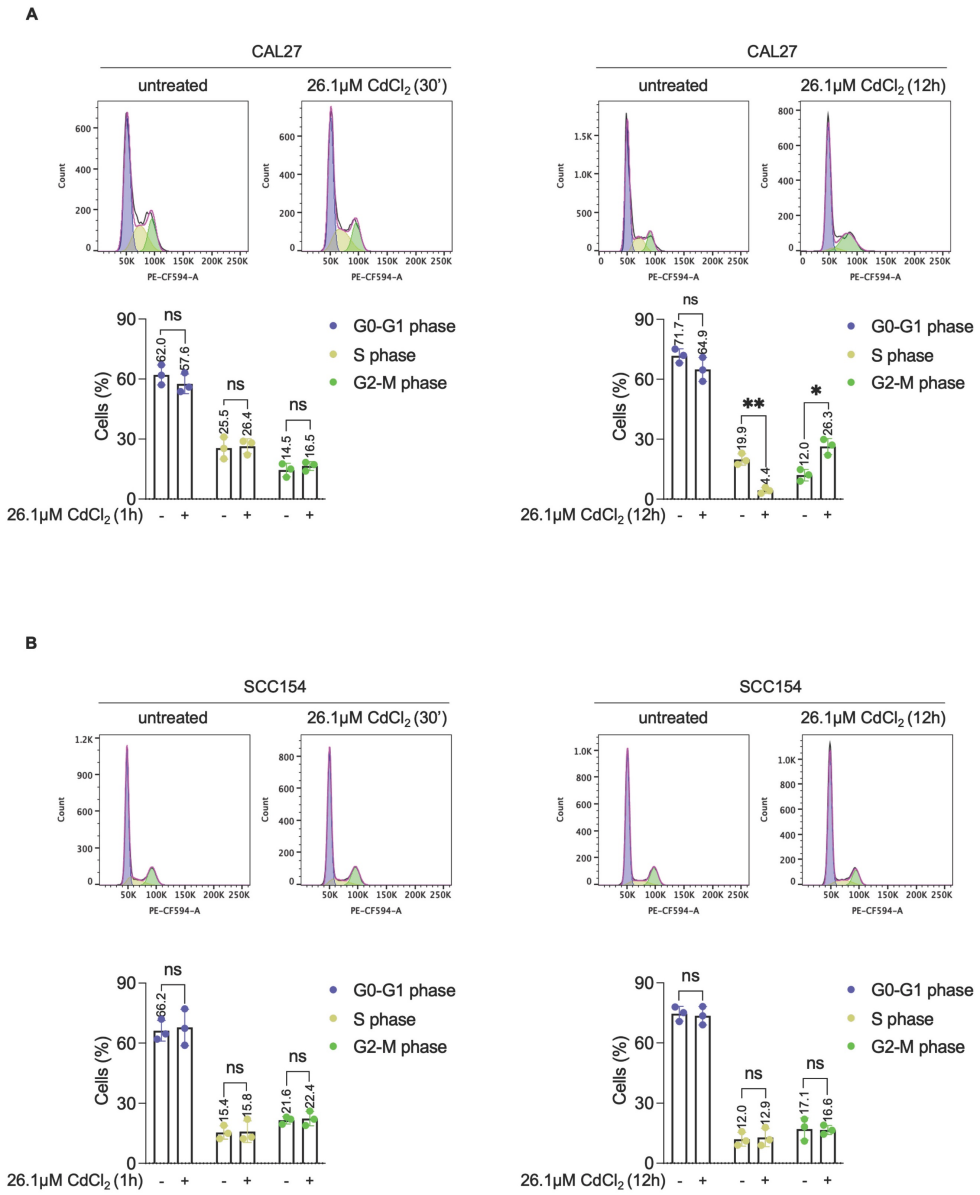
** CdCl_2_ exposure induces G2-M phase arrest in CAL27 cells derived from never smokers.** Cell cycle analysis via flow cytometry and relative histograms of CAL27 **(A)** and SCC154 **(B)** cells treated with 26.01μM CdCl_2_ for 1h and 12h. Each experiment was performed in triplicate. Histograms are presented as mean ± SD. *p*-value: *≤0.05; **≤0.01. ns: not significant.

To elucidate the molecular mechanisms underlying the differential sensitivity and tolerance to Cd^2+^ between CAL27 and SCC154 OSCC cell lines, we performed a comparative proteomic analysis following 12h exposure to 26.1μM CdCl_2_. A total of 222 differentially expressed proteins (DEPs) were identified in CdCl_2_-treated CAL27 cells compared to untreated controls, with 90 upregulated and 132 downregulated proteins (FDR: 0.05) (Figure 6A; File S1). GSEA of the top 20 upregulated and the top 20 downregulated proteins in CAL27 cells revealed significant enrichment of biological processes related to *response to reactive oxygen species*, *cellular response to hypoxia*, *intracellular iron ion homeostasis,* and *negative regulation of apoptotic signaling pathway* (Figure 6C). In contrast, the number of DEPs identified in CdCl_2_-treated SCC154 cells compared to controls was limited to six; these included three upregulated proteins - heme oxygenase 1 (HMOX1), solute carrier family 30 member 1 (SLC30A1), and MT2A - and three downregulated proteins - Four and A Half LIM Domains 2 (FHL2), UTP18 Small Subunit Processome Component (UTP18), Hemoglobin Subunit Alpha 2 (HBA2) (FDR: 0.05) (Figure 6B). Gene Ontology of these DEPs in SCC154 cells showed significant enrichment for pathways involved in *response to cadmium ion* and *detoxification of inorganic compounds* (Table S1. Among the top 20 downregulated proteins in CAL27 cells was the iron-dependent ribonucleotide reductase M2 (RRM2), a key enzyme involved in DNA synthesis. RRM2 suppression is consistent with iron depletion and was accompanied by upregulation of hypoxia inducible factor-1 alpha (HIF-1α), a known sensor of cellular iron and oxygen levels. Given that RRM2 suppression is associated with impaired DNA repair and genome instability 67,68, we hypothesized that CdCl_2_-induced iron depletion might contribute to DNA damage in CAL27 cells. Supporting this hypothesis, flow cytometry assays revealed a marked increase in the percentage of pγ-H2AX^+^ CAL27 cells (mean %, CAL27^untreated^: 1.91 vs. CAL27^26.1μM CdCl2^: 37.0) following 12h treatment with CdCl_2_ treatment (Figure 6D). Notably, this was accompanied by a significant reduction of S-phase (mean %, CAL27^untreated^: 19.9 vs. CAL27^26.1μM CdCl2^: 4.4) and an accumulation of G2-phase CAL27 population (mean %, CAL27^untreated^: 12.0 vs. CAL27^26.1μM CdCl2^: 26.3) (Figure 7A). These effects were not observed in SCC154 cells (Figure 6D, Figure 7B).

### Chronic exposure to CdCl_2_ induces adaptive tolerance in CAL27 cells

**Figure 8:**
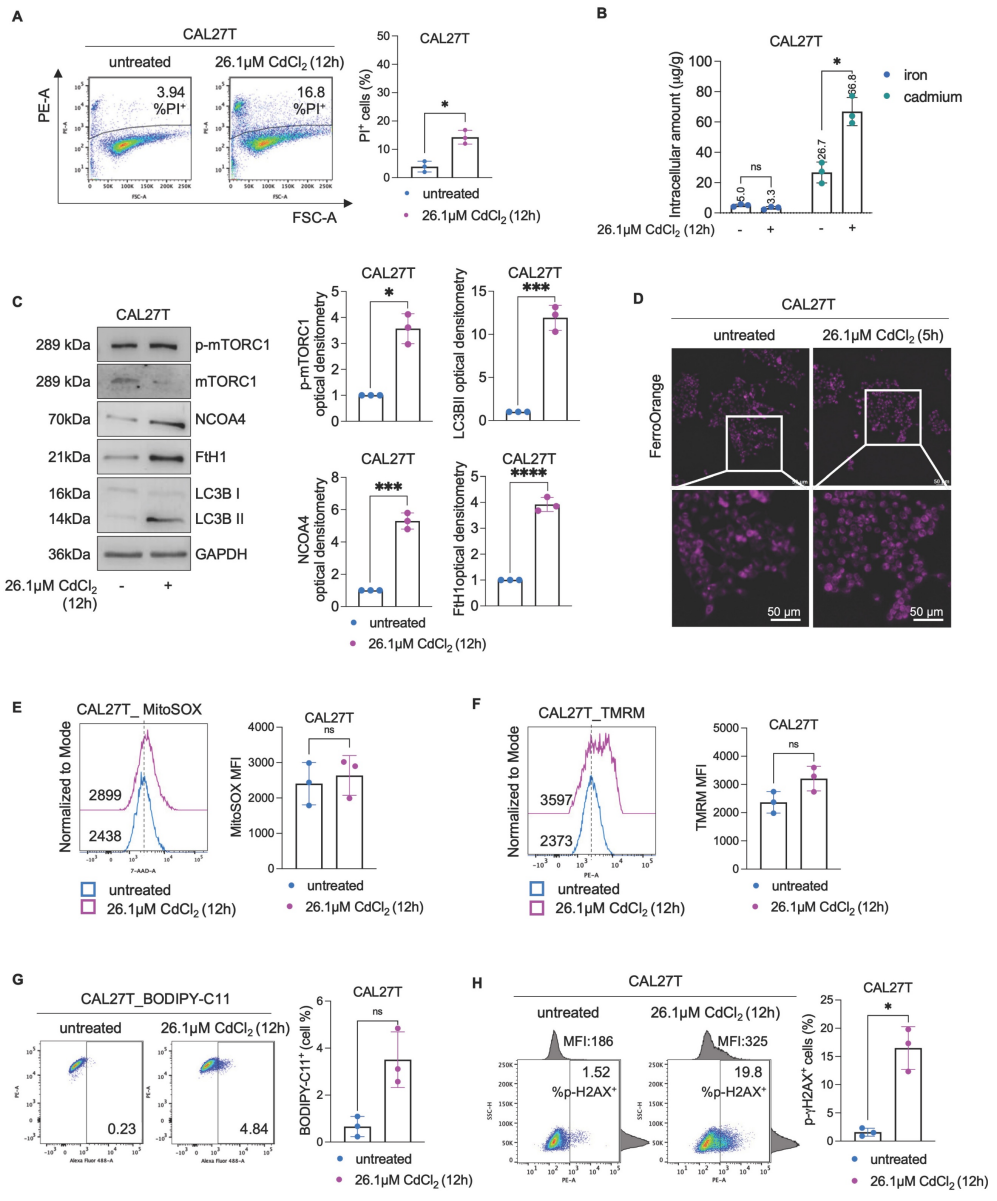
**Effects of CdCl_2_ exposure in CAL27T cells.** To obtain CAL27T (CAL27 Tolerant to Cd) CAL27 were exposed to low doses of CdCl_2_ (10μM) for 30 days. **(A)** PI flow cytometry assay and relative histograms of CAL27T cells treated with CdCl_2_ (26.01μM for 12h). % of dead cells (PI positive) are reported in each dot plot. **(B)** Quantification of iron and cadmium intracellular amount through ICP-MS in CAL27T cells treated with 26.01μM CdCl_2_ (12h).** (C)** Western blot analysis and relative optical densitometry of NCOA4, FtH1, LC3B (I-II), mTORC1 and p-mTORC1 in CAL27T cells treated with 26.01μM CdCl_2_ (12h). GAPDH was used as normalization control for protein quantification.** (D)** Fluorescence microscopy analysis of LIP content with FerroOrange dye in CAL27T cells after treatment with 26.01μM CdCl_2_ (12h). Flow cytometry analyses and relative histograms of mitochondrial ROS amount **(E)**, mitochondrial membrane potential **(F)** and lipid peroxidation **(G)** assessed by using MitoSOX, TMRM and BODIPY-C11 reagents, respectively, in CAL27T cells following treatment with 26.01μM CdCl_2_ (12h). Each experiment was performed in triplicate. Histograms are presented as mean ± SD. *p*-value: *≤0.05; ***≤0.001; ****≤0.0001. ns: not significant.

To investigate whether the prolonged exposure to low doses of Cd^2+^ can activate adaptive mechanisms promoting tolerance - as observed in SCC154 cells derived from a smoker patient -CAL27 cells were chronically exposed to 10 μM CdCl_2_ for 30 days (hereafter referred to as CAL27T). Following this treatment, CAL27T cells were exposed to 26.1μM CdCl_2_ for 12h. Noteworthy, the percentage of PI^+^ CAL27T cells was only 3.94% under basal condition and did not exceed 16.8% upon acute CdCl_2_ poisoning (Figure 8A). The capacity of CAL27T cells to accumulate Cd was also remarkably reduced. While the baseline intracellular Cd content was 26.7μg/g, Cd accumulation after 26.1μM CdCl_2_ exposure increased less than 3-fold reaching 66.8 μg/g. Furthermore, no variation in total intracellular iron levels was detected, suggesting the preservation of iron homeostasis (Figure 8B). Consistent with these findings, no evidence of ferritinophagy activation was observed in CAL27T cells. Although a slight increase in LC3BII levels was detected, markers of suppressed autophagy and restored iron storage, including p-mTORC1, NCOA4 and FTH1 protein levels, were all upregulated (Figure 8C). Moreover, the LIP, measured after 5h CdCl_2_ exposure (the time point at which LIP accumulation became evident in parental CAL27 cells), remained stable up to 12h (Figure 8D, Movies S5-6). In line with the attenuated iron dysregulation, HIF-1α remained unaltered Figure S5, only slight increase in mitoROS production and ΔѰ_m_ variation were observed (Figures 8E-F), and lipid peroxidation remained limited to 4.84% (Figure 8G). Finally, the extent of DNA damage in CAL27T reached a maximum of 19.8%, corresponding to approximately half of that detected in their parental CdCl_2_- sensitive counterparts (Figure 8H).

## Discussion

Oxidative stress plays a pivotal role in both Cd^2+^-induced toxicity and carcinogenesis. Acute Cd^2+^ exposure leads to enhanced production of ROS and consequent oxidative damage through multiple mechanisms, including depletion of antioxidant scavengers, interference with antioxidant enzymes, and mitochondrial dysfunction 69-71. As a result, various forms of cell death, such as necrosis, apoptotic-like cell death, autophagy, and ferroptosis, can be triggered by acute Cd^2+^ toxicity 72-75. In contrast, prolonged exposure to low levels of Cd^2+^ enables cells to activate adaptive responses, upregulating genes involved in redox homeostasis, such as *HMOX1*, *GSH*, and *MTs*, which mitigate oxidative stress and, at the same time, allow for a continued proliferation of damaged cells, contributing to carcinogenesis 13,24,76,77. The tipping point between adaptation and injury depends on multiple cellular and environmental factors.

In this study, we explored the effects of acute Cd^2+^ exposure in OSCC cells and uncovered a fascinating dichotomy: CAL27 cells (from never-smokers) are highly sensitive to Cd^2+^ cytotoxicity, while SCC154 cells (from smokers) exhibit a striking tolerance. This observation led us to investigate the interplay between Cd-mediated dysregulation of iron homeostasis, autophagy, and ferroptosis. The literature reports conflicting findings regarding the effects of Cd^2+^ in OSCC cells; Fan, T. *et al.* showed that repeated Cd^2+^ exposure promotes migration and invasion of CAL27 cells via ROS/NUPR1-dependent autophagy 36, while So, K.Y. *et al.* found that Cd^2+^ exposure reduces the catalase (CAT) expression, increases HMOX1 and triggers apoptosis in YD8 and YD10B oral cancer cells 78. Here, we found that in never-smoker-derived OSCC cells, vulnerability to Cd^2+^ toxicity is linked to the disruption of iron homeostasis. As a divalent metal cation, Cd^2+^ competes with iron for cellular uptake and utilization, potentially displacing it in key enzymes involved in respiration, metabolism, DNA synthesis and repair 79. The effects of this competition vary by cell types. In human Burkitt lymphoma BJAB cells, Cd^2+^ decreases intracellular free Fe^2+^ and cell viability 80, while in rat liver, kidney, and testicular cancer cells, Cd^2+^ increases LIP and promotes ROS formation via Fenton reactions 81,82. In CAL27 cells, we observed that Cd^2+^ first causes rapid iron depletion, followed by delayed LIP accumulation, likely via NCOA4-mediated ferritinophagy. NCOA4 acts as an autophagic receptor targeting ferritin for degradation and releasing stored iron in response to iron starvation 83-85. Importantly, iron supplementation reduces both Cd^2+^ accumulation and cytotoxicity, while *CD71* knockdown exacerbates Cd^2+^-induced cell death in CAL27 cells, likely impairing iron uptake. These results align with previous findings in other systems where increased intracellular iron protects against Cd^2+^-induced ROS and cytotoxicity 86. While we did not directly address the mechanisms of Cd^2+^ accumulation in CAL27 cells, our data suggest that 61-63 competition for CD71, a primary cellular iron transporter, could play a role. Indeed, CAL27 cells express higher CD71 levels and accumulate more Cd than SCC154 cells Figure S6A). Further studies are warranted.

Excessive ferritinophagy can trigger ferroptosis, the pioneer of the metals-induced RCD modes (65, caused by iron-dependent peroxidation of polyunsaturated fatty acids 87,88. While typically associated with iron, emerging evidence suggest that non-ferrous metals, such as Cd^2+^ can also induce ferroptosis by promoting ferritinophagy and iron overload 89-92. Here, we demonstrate that in CAL27 cells Cd^2+^-induced ferritinophagy leads to FtH1 degradation, increased LIP, mitochondrial ROS production, mitochondrial membrane hyperpolarization, disruption of GSH/GPX4 antioxidant system, lipid peroxidation, and ultimately ferroptosis. Interestingly, *NCOA4* knockdown in CAL27 cells did not prevent CdCl_2_-induced cell death; instead, it activated the IRP system and upregulated CD71, potentially promoting iron uptake, and maintaining ferroptosis susceptibility. Pharmacological inhibition of lipid peroxidation by using Fer-1 reduced Cd^2+^-cytotoxicity but not mitochondrial dysfunction, while autophagy inhibition by Baf mitigated both lipid peroxidation and mitochondrial damage, suggesting that ferritinophagy-mediated mitoROS may be an upstream event in Cd^2+^-induced ferroptosis. However, contributions from other forms of autophagy cannot be excluded.

In contrast, OSCC cells derived from smokers (SCC154), display high baseline antioxidant capacity, including elevated FtH1, GPX4, MT2A, and HMOX1, consistent with chronic adaptation to Cd exposure via tobacco use. MT2A detoxifies Cd and scavenges ROS 93, while HMOX1, a stress-inducible enzyme, is upregulated in response to oxidative stress and facilitates heme degradation 78,94. Notably, both CAL27 and SCC154 upregulate HMOX1 following CdCl_2_ exposure, but only SCC154 cells are protected from ferroptosis, likely due to more effective antioxidant and iron-handling mechanisms. Factors such as differential regulation of ferritinophagy, GSH levels, and lipid repair pathways may contribute to the observed cell-type-specific responses. GSH levels also increase in SCC154 cells after Cd^2+^ exposure further supporting their enhanced resistance. Extending these observations to clinical specimens, we found that *HMOX1, MT2A,* and* FtH1* were significantly overexpressed in OSCC tissues derived from 7 OSCC smoker patients compared to 7 OSCC never-smoker patients (Figure S6B), supporting the *in vivo* relevance of our findings.

CAL27T cells - generated by chronic low dose of Cd exposure, acquire tolerance to acute CdCl_2_ treatment, do not undergo ferritinophagy or ferroptosis, and do not show iron depletion or HIF-1α overexpression, unlike parental CAL27 cells. These data suggest that HIF-1α stabilization is not a general response to CdCl_2_ exposure but is instead linked to iron-dependent stress in sensitive cells, potentially contributing to Cd cytotoxicity.

Beyond immediate toxicity, our results reveal that Cd^2+^ exposure has long-term effects: in sensitive CAL27 cells, Cd^2+^-induced iron depletion and oxidative stress downregulate RRM2, reduce S-phase entry, increase G2 accumulation, and elevate γH2AX, indicating DNA damage. Given the reduced proportion of cells in S-phase, we propose this damage is not solely replication-dependent, but may arise from oxidative injury, impaired DNA repair, or checkpoint failure with significant implications for oral cancer progression.

In summary, our findings provide new insights into the mechanism of Cd^2+^ cytotoxicity in OSCC cells, revealing striking differences between cells from never-smokers and smokers. Sensitivity in non-smokers-derived cells is linked to autophagic ferroptosis and disrupted iron homeostasis while smokers-derived cells exhibit resistance through upregulated antioxidant defenses and metal detoxification. Chronic exposure to Cd^2+^, as experienced by smokers, induces adaptive responses that mitigate toxicity but may also foster cancer development through persistent cellular stress and genetic instability. These findings underscore the need to consider individual exposure histories when assessing environmental risks and highlight the importance of further studies to clarify mechanisms of Cd^2+^ accumulation, adaptive resistance, and long-term consequences in OSCC.

## Supplementary Material

Supplementary figures and tables.

Movies S1-S6, File S1.

## Figures and Tables

**Table 1:** Operating conditions and acquisition parameters for ICP-MS

Parameter	Setting
RF power (W)	500-1700
Reflected power	< 10
Plasma gas flow (L min^-1^)	15
Nebulizer gas flow (L min^-1^)	1,00
Auxiliary gas flow (L min^-1^)	0,80
He mode	collision cell mode
He gas flow (ml min^-1^)	5,00
Octopole bias (CCT bias) (V)	-21
Quadrupole bias (pole bias) (V)	-18

## Abbreviation

ROS: reactive oxygen species
RCD: regulated cell death
Fe²⁺: iron
Cu²⁺: copper
Ca²⁺: calcium
Zn²⁺: zinc
Mn²⁺: manganese
Cd²⁺: cadmium
GSH: glutathione
IARC: international agency for research on cancer
Nrf2: nuclear factor erythroid 2-related factor 2
AP-1: activator protein 1
NF-kB: nuclear factor-kB
MAPKs: mitogen-activated protein kinases
OSCC: oral squamous cell carcinoma
HNSCC: head and neck squamous cell carcinoma
MT2A: metallothionein 2A
NCOA4: nuclear receptor coactivator 4
FBS: fetal bovine serum
CdCl_2_: cadmium chloride
Fer-1: ferrostatin-1
Baf: bafilomycin
CAL27T: CAL27 tolerant
PI: iodide propidium
FtH1: ferritin heavy subunit
GPX4: glutathione peroxidase 4
MFRN1: mitoferrin 1
mTORC1: mechanistic target of rapamycin complex 1
p-mTORC1: phosphorylated mTORC1
LC3B: microtubule associated protein 1 light chain 3B
GAPDH: glyceraldehyde 3-phosphate dehydrogenase
TEM: transmission electron microscopy
LIP: labile iron pool
ΔѰ_m_: mitochondrial membrane potential
mitoROS: mitochondrial ROS
TMRE: tetramethylrhodamine ethyl ester
ICP-MS: inductively coupled plasma mass spectrometry
HNO_3_: nitric acid
CD71: transferrin receptor
LC-MS/MS: liquid chromatography tandem mass spectrometry
DDT: dithiothreitol
IAA: iodoacetamide
PAC: protein aggregation capture
CAN: acetonitrile
pγ-H2AX^+^: phospho-γ H2A histone family member X
DIA: data-independent acquisition
DEPs: differentially expressed proteins
HMOX1: heme oxygenase 1
SLC30A1: solute carrier family 30 member 1
FHL2: four and a half LIM domains 2
UTP18: small subunit processome component
HBA2: hemoglobin subunit alpha 2
RRM2: ribonucleotide reductase M2
HIF-1α: hypoxia inducible factor-1 alpha
NUPR1: nuclear protein 1
CAT: antioxidant enzyme catalase
IRP1: iron regulatory protein 1
