# Supplementary material for: Acute Exposure to Cadmium Triggers NCOA4-Mediated Ferritinophagy and Ferroptosis in Never-Smokers Oral Cancer Cells
Source: Int J Biol Sci. 2025 Jun 20;21(9):4131–52. doi: 10.7150/ijbs.111228 (PMC12223766; doi:10.7150/ijbs.111228)
Supplement: Supplementary file 1 — Supplementary figures and tables. [file ijbsv21p4131s1.pdf]

Table S1. Gene Ontology (GO) of DEPs in SCC154 cells treated with 26.01µM $CdCl_2$ (12h) *vs* untreated.

GO-term	description	count in network	strength	signal	false discovery rate
GO:0046686	Response to cadmium ion	3 of 59	2.22	1.32	0.0092
GO:0046916	Cellular transition metal ion homeostasis	3 of 108	1.96	0.99	0.0269
GO:0097501	Stress response to metal ion	2 of 18	2.56	0.98	0.0345
GO:0061687	Detoxification of inorganic compound	2 of 17	2.59	0.98	0.0345
GO:0098754	Detoxification	3 of 132	1.87	0.93	0.0324
GO:0010035	Response to inorganic substance	4 of 532	1.39	0.8	0.0324

Figure S1. Cell viability and migration capacity of OSCC cells upon $CdCl_2$ exposure. A. Cell viability assay of CAL27, OT1109, SCC154 and SCC090 upon treatment with 0.1, 1, 10, 50 and 100µM of $CdCl_2$ (12h) and relative IC50 values. **B.** MTT assay of SCC154 cells after treatment with growing concentration of $CdCl_2$ (0.1, 1, 5 and 10µM) at T0,12h and 24h. **C.** Representative images of a wound healing assay for SCC154 cells following treatment with increasing concentrations of $CdCl_2$ (0.1, 1, 5, and 10 µM) at time points T0, 12h, and 24h (10x magnification). The relative histogram displays the mean of the gap area of three biological replicates.

Figure S2. Fer-1 partially reverts mitochondrial dysfunction and lipid peroxidation induced by $CdCl_2$ only in CAL27 cells. Flow cytometry analyses and relative histograms of mitochondrial ROS amount (**A**), mitochondrial membrane potential (**B**) and lipid peroxidation (**C**) assessed by using MitoSOX, TMRM and BODIPY-C11 reagents, respectively, in CAL27 and SCC154 cells following treatment with 26.01µM $CdCl_2$ (12h) alone or pre-treated with Fer-1 (100µM for 24h). All data represent the mean of three independent experiments. Histograms are reported as mean ± SD. *p*-value: *≤0.05; **≤0.01. ns: not significant.

Figure S3. Baf partially reverts lipid peroxidation and mitochondrial dysfunction mediated by $CdCl_2$ only in CAL27 cells. Flow cytometry analyses and relative histograms of mitochondrial ROS amount (**A**), mitochondrial membrane potential (**B**) and lipid peroxidation (**C**) assessed by using MitoSOX, TMRM and BODIPY-C11 reagents, respectively, in CAL27 and SCC154 cells following treatment with 26.01µM $CdCl_2$ (12h) alone or in combination with Baf (1µM for 12h). Each experiment was performed in triplicate. Histograms are presented as mean ± SD. *p*-value: *≤0.05. ns: not significant.

Figure S4. Effects of *NCOA4* and *CD71* knockdown in CAL27 cells upon $CdCl_2$ exposure. A. Realtime PCR analysis of *NCOA4* in CAL27 cells treated or not with 26.01µM $CdCl_2$ upon *NCOA4* silencing (48h). **B**. Western blot analysis and relative optical densitometry of FtH1, CD71 and IRP1 in CAL27 cells either untreated or treated with 26.01µM $CdCl_2$ following *NCOA4* knockdown. GAPDH was used as normalization control for protein quantification. **C**. Representative dot plot (left) and relative histograms (right) of PI flow cytometry assay of CAL27 cells upon *NCOA4* silencing (48h), treated or not with 26.01µM $CdCl_2$. **D.** Realtime PCR analysis of *CD71* in CAL27 and SCC154 cells upon *CD71* silencing (24h). **E**. Representative dot plot (left) and relative histograms (right) of PI flow cytometry assay of CAL27 and SCC154 cells upon CD71 silencing (24h), treated or not with 26.01µM $CdCl_2$. Experiments were performed in triplicate. Histograms are presented as mean ± SD. *p*-value: *≤0.05; **≤0.01. ns: not significant.

Figure S5. Effect of $CdCl_2$ exposure on HIF-1α protein levels in OSCC cells. Western blot analysis and relative optical densitometry of HIF-1α in CAL27, SCC154 and CAL27T after treatment with with 26.01µM $CdCl_2$ (12h). GAPDH was used as normalization control for protein quantification. Each experiment was performed in triplicate. Histograms are presented as mean ± SD. *p*-value: *≤0.05. ns: not significant.

Figure S6. A. Flow citometry analysis and relative histograms of CD71 surface expression in CAL27, SCC154 and CAL27T at basal level. **B.** Box plots showing gene expression analysis of *HMOX1*, *MT2A*, *FtH1* and *GPX4* in OSCC patients classified as smokers and no-smokers. *p*-value: **≤0.01; ***≤0.001. ns: not significant.

Movie S1. Time lapse of fluorescence microscopy analysis of LIP content with FerroOrange dye in CAL27 untreated (12h) (10x magnification).

Movie S2. Time lapse of fluorescence microscopy analysis of LIP content with FerroOrange dye in CAL27 after treatment with 26.01µM $CdCl_2$ (12h) (10x magnification).

Movie S3. Time lapse of fluorescence microscopy analysis of LIP content with FerroOrange dye in SCC154 untreated (12h) (10x magnification).

Movie S4. Time lapse of fluorescence microscopy analysis of LIP content with FerroOrange dye in SCC154 after treatment with 26.01µM $CdCl_2$ (12h) (10x magnification).

Movie S5. Time lapse of fluorescence microscopy analysis of LIP content with FerroOrange dye in CAL27T untreated (12h) (10x magnification).

Movie S6. Time lapse of fluorescence microscopy analysis of LIP content with FerroOrange dye in CAL27T after treatment with 26.01µM $CdCl_2$ (12h) (10x magnification).

Figure S1

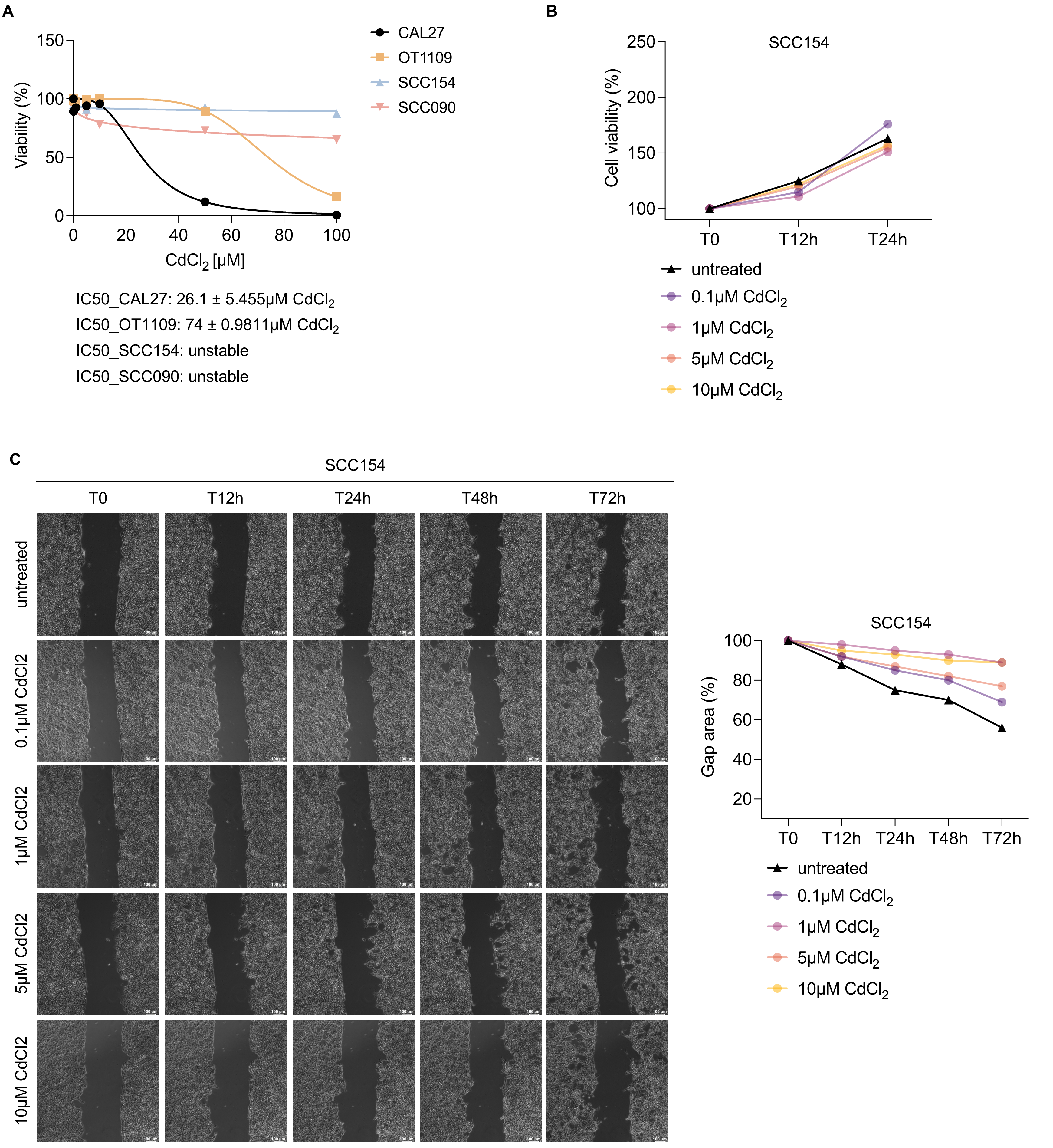
A
CAL27
OT1109
SCC154
SCC090
Viability (%)
CdCl2 [μM]
IC50_CAL27: 26.1 ± 5.455μM CdCl2
IC50_OT1109: 74 ± 0.9811μM CdCl2
IC50_SCC154: unstable
IC50_SCC090: unstable
B
SCC154
Cell viability (%)
T0
T12h
T24h
untreated
0.1μM CdCl2
1μM CdCl2
5μM CdCl2
10μM CdCl2
C
SCC154
T0
T12h
T24h
T48h
T72h
untreated
0.1μM CdCl2
1μM CdCl2
5μM CdCl2
10μM CdCl2
SCC154
Gap area (%)
T0
T12h
T24h
T48h
T72h
untreated
0.1μM CdCl2
1μM CdCl2
5μM CdCl2
10μM CdCl2

Figure S2

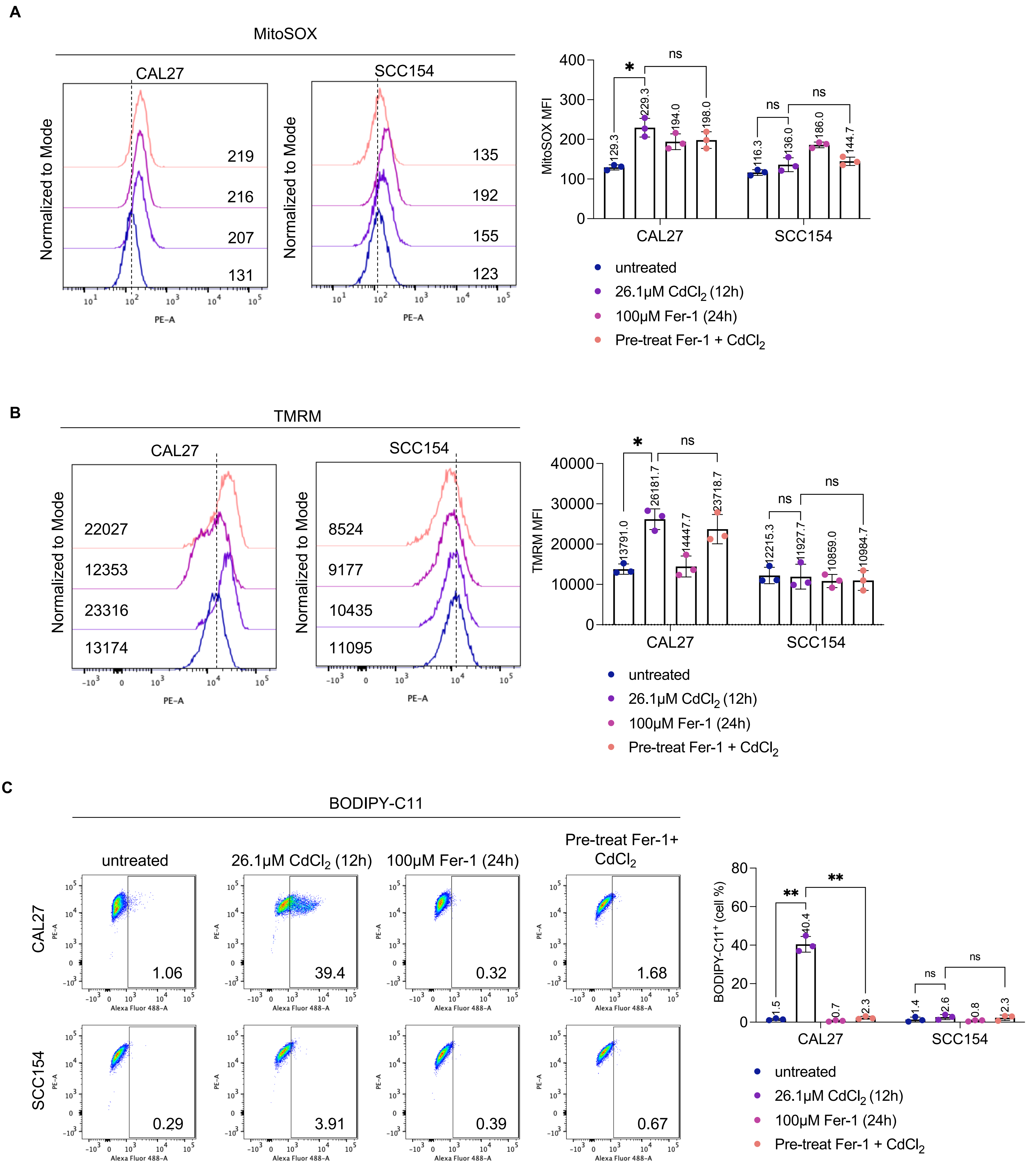
A
MitoSOX
CAL27
SCC154
Normalized to Mode
219
216
207
131
135
192
155
123
PE-A
MitoSOX MFI
129.3
229.3
194.0
198.0
116.3
136.0
186.0
144.7
ns
CAL27
SCC154
untreated
26.1μM CdCl2 (12h)
100μM Fer-1 (24h)
Pre-treat Fer-1 + CdCl2
B
TMRM
22027
12353
23316
13174
8524
9177
10435
11095
TMRM MFI
13791.0
26181.7
14447.7
23718.7
12215.3
11927.7
10859.0
10984.7
C
BODIPY-C11
untreated
26.1μM CdCl2 (12h)
100μM Fer-1 (24h)
Pre-treat Fer-1+ CdCl2
1.06
39.4
0.32
1.68
0.29
3.91
0.39
0.67
Alexa Fluor 488-A
BODIPY-C11+ (cell %)
1.5
40.4
0.7
2.3
1.4
2.6
0.8
2.3

Figure S3

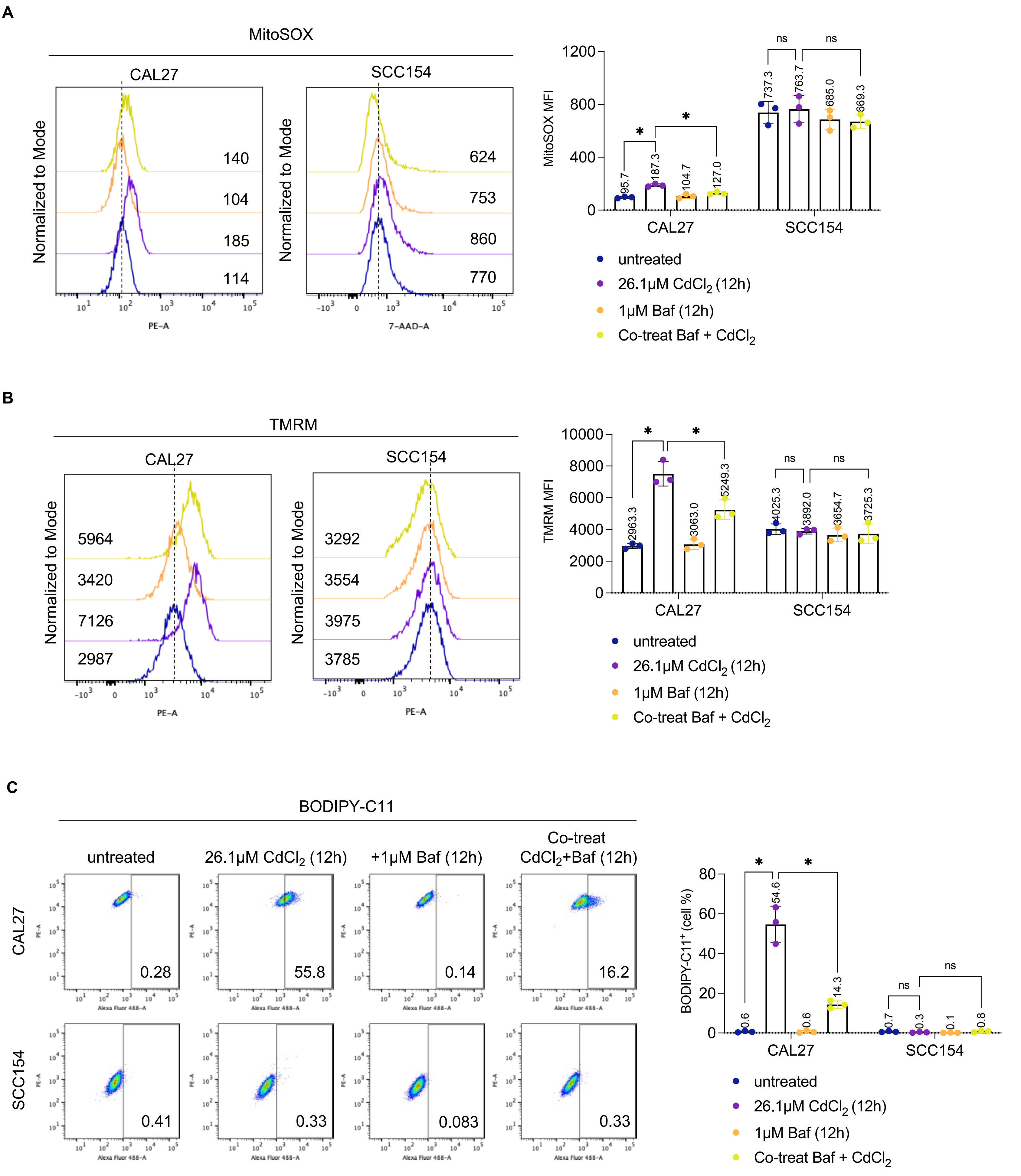
A
MitoSOX
CAL27
SCC154
Normalized to Mode
140
104
185
114
624
753
860
770
PE-A
7-AAD-A
MitoSOX MFI
1200
800
400
0
95.7
187.3
104.7
127.0
737.3
763.7
685.0
669.3
*
*
ns
ns
CAL27
SCC154
untreated
26.1μM CdCl2 (12h)
1μM Baf (12h)
Co-treat Baf + CdCl2
B
TMRM
CAL27
SCC154
Normalized to Mode
5964
3420
7126
2987
3292
3554
3975
3785
PE-A
TMRM MFI
10000
8000
6000
4000
2000
0
2963.3
3063.0
5249.3
4025.3
3892.0
3654.7
3725.3
*
*
ns
ns
CAL27
SCC154
untreated
26.1μM CdCl2 (12h)
1μM Baf (12h)
Co-treat Baf + CdCl2
C
BODIPY-C11
untreated
26.1μM CdCl2 (12h)
+1μM Baf (12h)
Co-treat
CdCl2+Baf (12h)
CAL27
SCC154
0.28
55.8
0.14
16.2
0.41
0.33
0.083
0.33
PE-A
Alexa Fluor 488-A
BODIPY-C11+ (cell %)
80
60
40
20
0
0.6
54.6
0.6
14.3
0.7
0.3
0.1
0.8
*
*
ns
ns
CAL27
SCC154
untreated
26.1μM CdCl2 (12h)
1μM Baf (12h)
Co-treat Baf + CdCl2

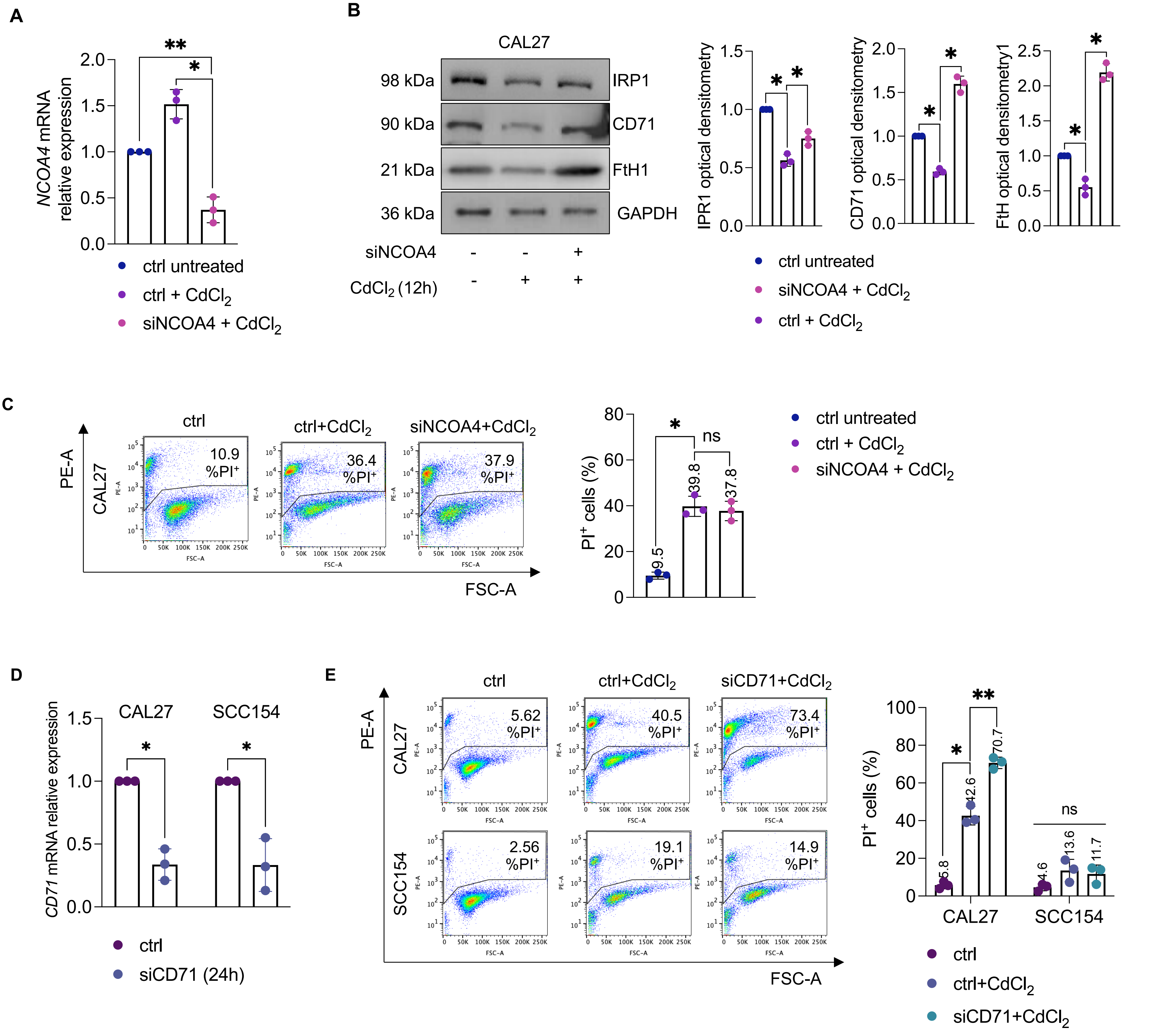
A
NCOA4 mRNA relative expression
2.0
1.5
1.0
0.5
0.0
**
*
ctrl untreated
ctrl + CdCl2
siNCOA4 + CdCl2
B
CAL27
98 kDa
IRP1
90 kDa
CD71
21 kDa
FtH1
36 kDa
GAPDH
siNCOA4 - - +
CdCl2 (12h) - + +
IPR1 optical densitometry
CD71 optical densitometry
FtH optical densitometry1
ctrl untreated
siNCOA4 + CdCl2
ctrl + CdCl2
C
ctrl
ctrl+CdCl2
siNCOA4+CdCl2
PE-A
CAL27
10.9 %PI+
36.4 %PI+
37.9 %PI+
FSC-A
PI+ cells (%)
9.5
39.8
37.8
ns
ctrl untreated
ctrl + CdCl2
siNCOA4 + CdCl2
D
CD71 mRNA relative expression
CAL27
SCC154
ctrl
siCD71 (24h)
E
ctrl
ctrl+CdCl2
siCD71+CdCl2
PE-A
CAL27
SCC154
5.62 %PI+
40.5 %PI+
73.4 %PI+
2.56 %PI+
19.1 %PI+
14.9 %PI+
FSC-A
PI+ cells (%)
5.8
42.6
70.7
4.6
13.6
11.7
ns
CAL27
SCC154
ctrl
ctrl+CdCl2
siCD71+CdCl2

Figure S5

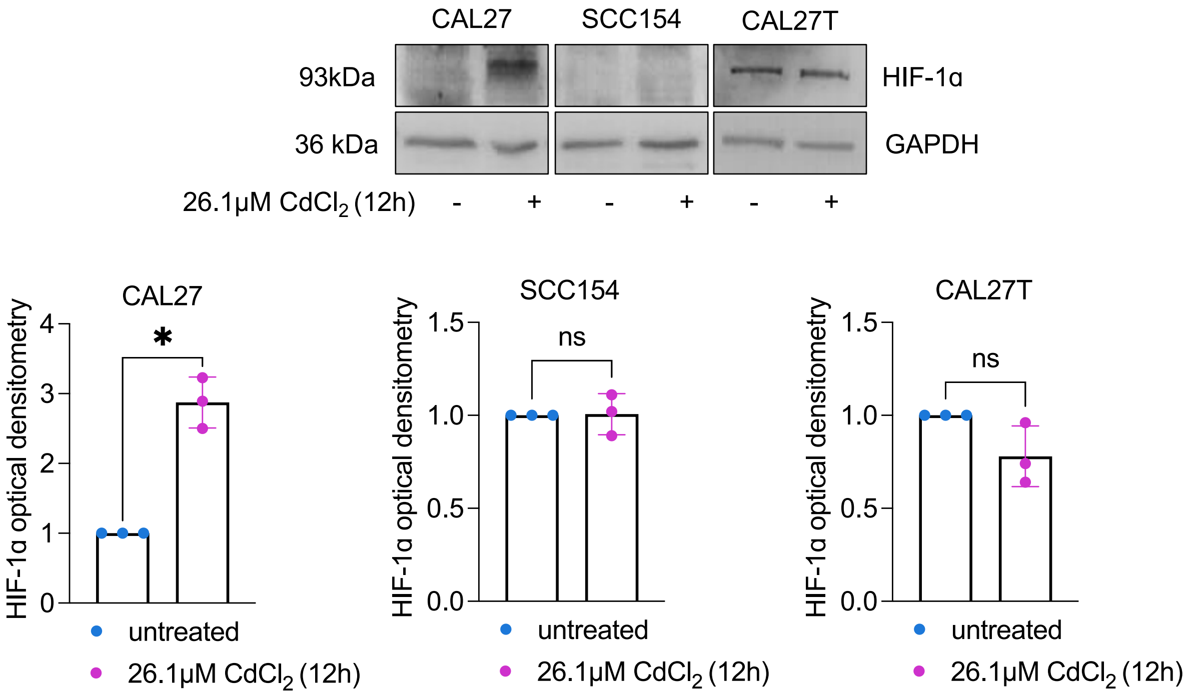
CAL27
SCC154
CAL27T
93kDa
HIF-1α
36 kDa
GAPDH
26.1μM $CdCl_2$ (12h)
- + - + - +
CAL27
HIF-1α optical densitometry
*
untreated
26.1μM $CdCl_2$ (12h)
SCC154
ns
HIF-1α optical densitometry
untreated
26.1μM $CdCl_2$ (12h)
CAL27T
ns
HIF-1α optical densitometry
untreated
26.1μM $CdCl_2$ (12h)

Figure S6

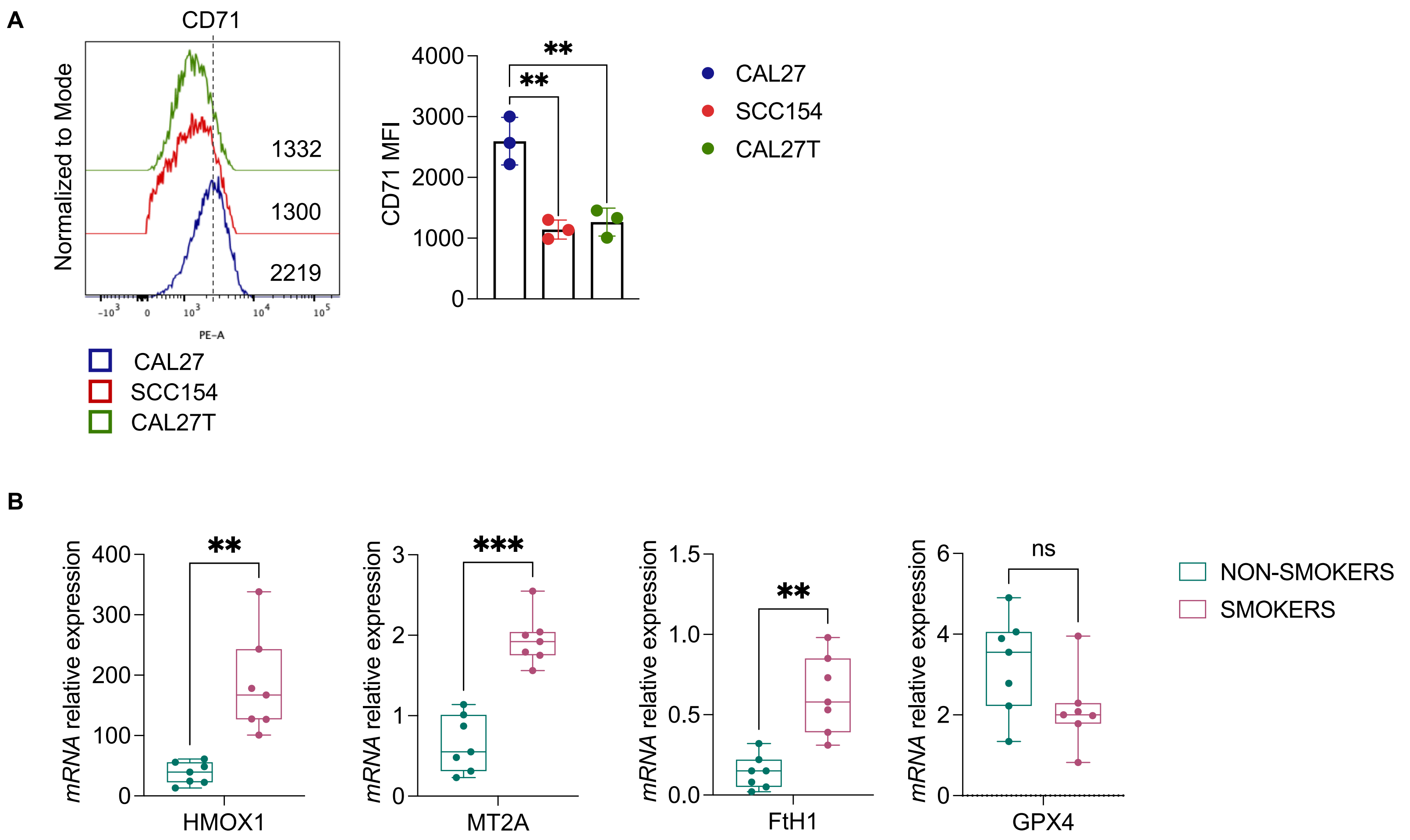
A
CD71
Normalized to Mode
1332
1300
2219
PE-A
CAL27
SCC154
CAL27T
CD71 MFI
4000
3000
2000
1000
0
**
**
CAL27
SCC154
CAL27T
B
mRNA relative expression
HMOX1
MT2A
FtH1
GPX4
**
***
**
ns
NON-SMOKERS
SMOKERS
